# Graftable SCoMPIs enable the labeling and X-ray fluorescence imaging of proteins†
†Electronic supplementary information (ESI) available: Additional studies, experimental procedures, and compounds characterization. See DOI: 10.1039/c8sc00886h


**DOI:** 10.1039/c8sc00886h

**Published:** 2018-04-13

**Authors:** Sarah Hostachy, Marie Masuda, Takayuki Miki, Itaru Hamachi, Sandrine Sagan, Olivier Lequin, Kadda Medjoubi, Andrea Somogyi, Nicolas Delsuc, Clotilde Policar

**Affiliations:** a Laboratoire des Biomolécules, LBM , Département de Chimie , École Normale Supérieure , PSL University , Sorbonne Université , CNRS , 75005 Paris , France . Email: nicolas.delsuc@ens.fr; b Department of Synthetic Chemistry and Biological Chemistry , Graduate School of Engineering , Kyoto University , Kyoto 615-8510 , Japan; c Sorbonne Université , École Normale Supérieure , PSL University , CNRS , Laboratoire des Biomolécules, LBM , 75005 Paris , France; d Nanoscopium Synchrotron SOLEIL Saint-Aubin , 91192 , Gif-sur-Yvette Cedex , France

## Abstract

Bio-imaging techniques alternative to fluorescence microscopy are gaining increasing interest as complementary tools to visualize and analyze biological systems. Among them, X-ray fluorescence microspectroscopy provides information on the local content and distribution of heavy elements (*Z* ≥ 14) in cells or biological samples. In this context, similar tools to those developed for fluorescence microscopy are desired, including chemical probes or tags. In this work, we study rhenium complexes as a convenient and sensitive probe for X-ray fluorescence microspectroscopy. We demonstrate their ability to label and sense exogenously incubated or endogenous proteins inside cells.

## Abstract 2

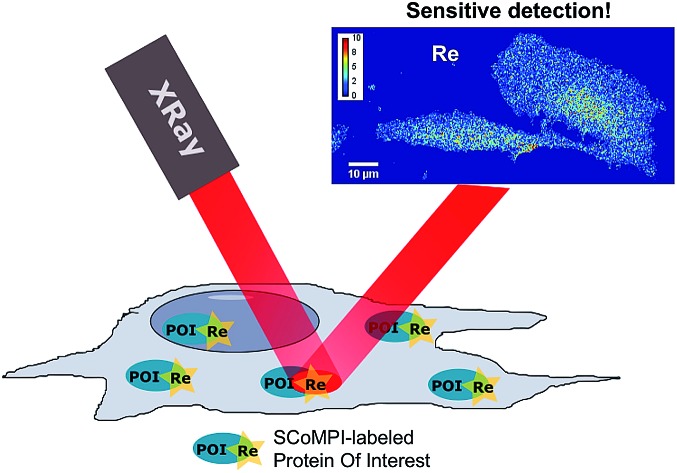
Sensitive detection of proteins by X-ray fluorescence microspectroscopy using the Re core of a single core multimodal probe for imaging.

## 

Bio-imaging enables the direct visualization of labeled biomolecules of interest, offering valuable insights into biological processes. Fluorescence microscopy is widely used and offers numerous advantages, including good spatial resolution (∼10^2^ × 10^2^ nm^2^) and availability of numerous probes and tags with high quantum yield and diverse spectroscopic properties. However, fluorescence techniques suffer from some drawbacks such as photobleaching. Quantum yields are dependent on the environment, with large variations upon polarity or possible quenching at high concentrations.^1^–^4^ For that reason, absolute quantification using classical fluorescence can be tricky.

Complementary methods for bio-imaging are now emerging. Microspectroscopies that provide simultaneous information on the local cellular environment can be of particular interest. Recently, vibrational techniques such as infrared microspectroscopies have become more popular, providing spatially resolved chemical information.^5^–^7^ Synchrotron-based X-ray fluorescence (SXRF) and absorbance microspectroscopies have also been used for the detection of metal centers in biological samples.^8^ Using synchrotron based X-fluorescence microspectroscopy (SXRF-MS), the simultaneous collection of data for a large range of elements is carried out, provided their edge energy is lower than the incident energy. Metal-based probes can be imaged using this technique and information on their elemental environment is also immediately available. Moreover, the spatial resolution of this technique is in the 10 nm to 1 μm range, which is relevant for sub-cellular imaging. Finally, XRF is a quantitative technique: 2D-quantification is possible using commercially available standards.

Accessibility to new probes for this emerging technique would bolster its use for further investigation of biological mechanisms. Studies have been performed in this direction with the development of particles, quantum dots, polymers containing lanthanide and carbon nanotubes.^9^–^13^ However no graftable molecular tag suitable for protein imaging by XRF has been designed so far. Policar's group is strongly interested in the development and use of Re(i) tricarbonyl complexes as multimodal probes for fluorescence microscopy and infrared microspectroscopy.^14^,^15^ They display good biocompatibility and stability features, as well as unique spectroscopic properties, and they have been widely used for imaging using a single modality –mainly luminescence^16^,^17^ or as efficient bimodal probes in the mid-IR range and luminescence.^18^–^27^ In the present work, we have investigated the potential of these SCoMPIs (Single Core Multimodal Probes for Imaging) for a new modality, namely X-ray fluorescence. The rhenium center displays two intense signals at 8.6 and 10.28 keV, corresponding to its L-bands. Re has a very low natural abundance,^28^ which would ease the detection of the SCoMPI-labeled molecule with a high signal-to-noise ratio. Very recently, Massi and Harris have shown that Re complexes can be mapped at the single cell level by XRF.^29^ However, X-ray fluorescence molecular tags or probes that can be grafted onto target biomolecules and that do not modify to a large extent their physico-chemical properties are still needed.

In order to test the efficiency of Re(i) as a tag for SXRF, we wanted to assess whether low-abundant proteins could be labeled with SCoMPIs and mapped by SXRF-MS in a cellular environment. To do so, we explored two different approaches: (1) *in vitro* labeling of proteins that can be internalized in cells and (2) labeling of membrane-bound endogenous proteins in cells (Fig. 1a and b).

**Fig. 1:**
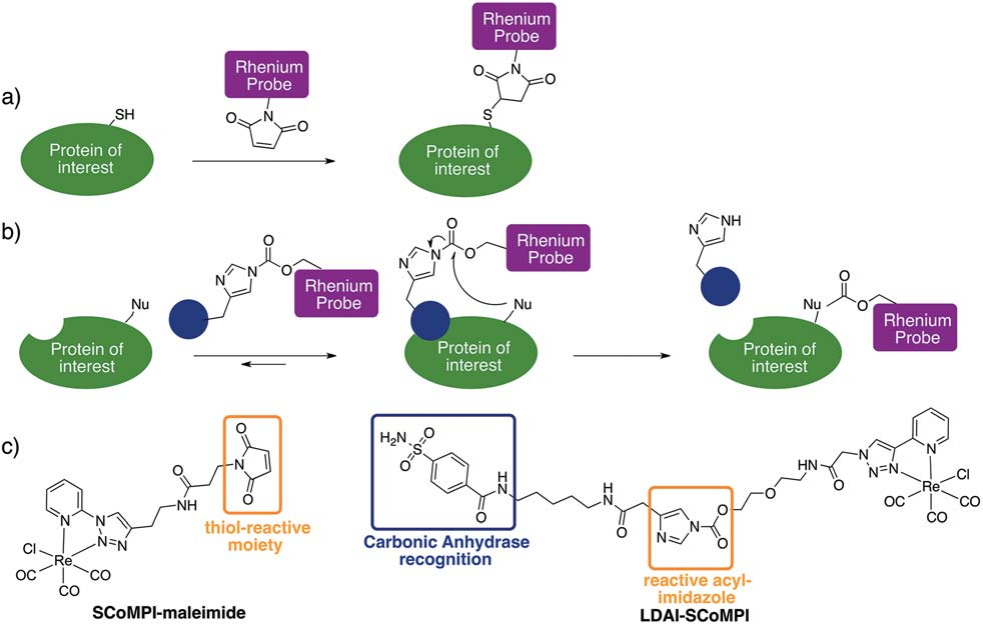
Labeling of proteins with SCoMPI for X-ray fluorescence imaging. (a) Labeling of an exogenous protein using the thiol maleimide reaction. (b) Labeling of an endogenous protein using the “Ligand-Directed Acyl Imidazole” chemistry. The blue circle represents a ligand of the protein. (c) (left) Structure of SCoMPI-maleimide for the *in vitro* labeling of homeodomains and (right) structure of LDAI-SCoMPI for the labeling of endogenous carbonic anhydrases.

For the first approach (Fig. 1a), we selected an *Engrailed* homeodomain. Homeodomains (HD) are domains of the highly conserved homeoproteins that are able to penetrate cells.^30^–^33^ The *Engrailed* homeodomain, in particular, has been used as a model to study some of the unconventional translocation mechanisms of homeodomains.^31^–^36^ In the present study, we chose to produce two constructions: (1) HD is *Engrailed* 2 homeodomain itself, and (2) NLS-HD displays an extended sequence with a putative Nuclear Localisation Signal (NLS) sequence. Neither HD nor NLS-HD bear a cysteine residue in their native sequence and for both constructions, a single cysteine was introduced at the N-terminus, for site-specific labeling of the protein through thiol–maleimide coupling (see ESI†). We also designed and synthesized a new SCoMPI bearing a maleimide moiety (Fig. 1c left).^37^,^38^ The synthetic pathway we used is general for SCoMPIs:^20^–^22^ a pyridine-1,2,3-triazole (pyta) ligand was first synthesized by copper(i)-catalyzed azide–alkyne cycloaddition (CuAAC) “click” chemistry between 2-ethynylpyridine and an azide with a relevant linker/function (here a Boc-protected amine). The reaction of this ligand with the Re(CO)_5_Cl precursor resulted in the formation of a Re(i) tricarbonyl complex. Finally, the amine was deprotected and reacted with an activated ester linked to the maleimide moiety (Scheme S1†).

A five-fold excess of the SCoMPI functionalized with a maleimide was used to ensure a complete reaction and both constructs could be efficiently labeled *in vitro* with this SCoMPI (see ESI†). Fluorescence and infrared properties of the labeled proteins were similar to those of the parent complex (Fig. S1 and S2†) showing that the SCoMPI was not modified during the labeling step. CHO cells were grown on Si_3_N_4_ slides that are commonly used for SXRF imaging, incubated in the presence of SCoMPI-labeled HD (Re-HD) or NLS-HD (Re-NLS-HD), washed and chemically fixed using an aqueous solution of paraformaldehyde (PFA). The chemical fixation is well suited in this case since the probe is covalently grafted to the proteins, which are immobilized by PFA fixation. Consequently, we assumed there is little risk of re-localization upon fixation.

None of the SCoMPI-labeled proteins could be detected by SR-FTIR microspectroscopy nor discriminated from the background signal of cells by fluorescence microscopy. But, as shown in Fig. 2, Re from the tags could be detected and mapped by SXRF-MS. Control samples did not display any Re signals in this region (data not shown). The SXRF modality of the SCoMPI is of interest as it lowered the detection limit of the SCoMPI labeled molecules, which validates Re-SCoMPI as sensitive probes for SXRF imaging. As expected for Re-HD, the signal was distributed in the whole cell. The Re-NLS-HD signal was also detected in the whole cell. However, as shown in the merged images (Fig. 2), comparison with the distributions of other biologically relevant metals (in particular Zn which is known to be abundant in the nucleus) showed that there is a slight accumulation of the NLS-HD construction in the nucleus. Moreover, Re-NLS-HD was significantly more internalized in cells, with an average increase by 4–5-fold of the uptake as compared to Re-HD (Table 1). The same trend was observed by MALDI mass spectrometry quantification of these constructs: NLS-HD was found to internalize 10-fold better than HD (Cardon *et al.* manuscript in preparation).

**Fig. 2:**
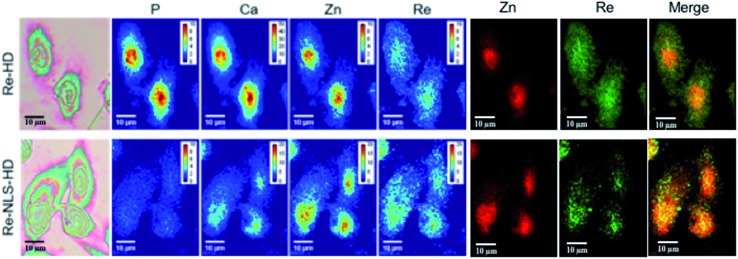
X-Ray fluorescence imaging of CHO cells incubated with Re-HD (top) and Re-NLS-HD (bottom). CHO cells were grown on Si_3_N_4_ slides for 24 h, incubated 1 h at 37 °C with 10 μM of labeled protein, washed, fixed and air-dried. The images from the left show the bright field image of the measured cells, the distribution of P, Ca, Zn, and Re measured by SXRF-MS with different color codes and Zn/Re merged images. Measurement conditions: 13.5 keV, beam size: 300 × 300 nm^2^, image pixel size: 300 nm. The XRF peak intensities were calculated by using the PyMca software,^42^ and are shown in counts per s. Images recorded at the Nanoscopium beamline (SOLEIL synchrotron, Paris) with a high intensity nanobeam of 300 × 300 nm^2^ size created by the Kirkpatrick–Baez focusing mirror of the beamline.^43^

**Table 1:** Semi-quantification of Re-labeled proteins in CHO or A549 cells

	Mean quantity/pixel^*a*^ (10^–21^ mol)	Quantity/cell^*a*^ (10^–18^ mol)
Re-HD (CHO)	1.95 (±0.03)	9.44 (±0.12)
Re-NLS-HD (CHO)	11.2 (±1.0)	43.88 (±22.4)
Re-LDAI-CA (A549)	1.79 (±0.17)	19.18 (±8.73)

^*a*^Mean of the values obtained for each cell of the image. Standard deviation in parenthesis (2 or 3 different cells).

Encouraged by these findings, we wanted to investigate whether endogenous proteins could also be detected by SXRF-MS using Re tags. We decided to use the “Traceless Affinity-Labeling” strategy developed by Hamachi's group, as it enables the modification of endogenous proteins without prior engineering of the protein of interest (POI) or major loss of protein function (Fig. 1b).^39^–^41^

Traceless labeling relies on the use of a ligand (inhibitor) of the POI, linked to a tag by a cleavable linker. Upon binding to the protein through ligand recognition, the cleavable linker is brought in close proximity to nucleophilic residues on the POI, which leads to the covalent binding of the tag to the protein. The so-called “Ligand-Directed Acyl Imidazole” (LDAI) chemistry was shown to have interesting features with regards to the reactivity and stability of the reagent in biological media.^44^,^45^ A LDAI reagent was designed and optimized for the labeling of human Carbonic Anhydrase I (hCA-I), a cytosolic isoform of Carbonic Anhydrase (CA).^46^ It was also used on A549 cells for the labeling of membrane-bound hCA-XII and, to a lesser extent, hCA-IX, which are known cancer markers. We chose to use this design to obtain a LDAI-SCoMPI reagent for the labeling of CA-IX and XII (Fig. 1c right).^47^

The convergent synthesis of LDAI reagents consisted of the synthesis of ligand-imidazole moieties and of a NHS-activated carbonate analog of the probe, which were then coupled, leading to the LDAI reagent (see ESI and Schemes S2–S4†). The synthesis of the benzenesulfonamide inhibitor-imidazole moiety (Fig. 1c) was carried out according to a published procedure,^46^ with slight modifications (see ESI†). We then examined its labeling efficiency towards purified hCA-I, a soluble isoform of carbonic anhydrase. A 10 μM solution hCA-I in HEPES was incubated with 2 or 20 equivalents of the LDAI reagents at 37 °C. The reaction was monitored for 6 h, analyzed either by MALDI-TOF MS or fluorescence imaging of SDS-PAGE gel migration for fluorescence detection of the SCoMPI (ESI†). A control containing a competitive inhibitor of hCA-I, (namely ethoxyzolamide, EZA, a stronger inhibitor than benzenesulfonamide) was also analyzed at 6 h (Fig. S4†). Because of the small size of the label relative to the protein (29 kDa), we assumed that the probe has a negligible influence on ionization properties. Thus, to estimate the labeling rate, the relative peaks area obtained from MALDI-TOF analysis of the labeled and of the unlabeled hCA-I was used. In the presence of 20 equivalents of LDAI-SCoMPI, a maximal labeling rate of hCA-1 (*ca.* 80%) was obtained within 2 h. In the presence of EZA, no labeling was observed. This confirms that the labeling is driven by the recognition of the ligand.

Carbonic anhydrases are overexpressed under hypoxic conditions. A549 cells were grown on Si_3_N_4_ slides, placed under hypoxic conditions for 24 h, and then incubated with 10 μM of LDAI-SCoMPI. After washing of the cells to remove the unreacted LDAI-SCoMPI, cells were fixed with PFA and mapped using XRF imaging. This technique provides a projected image (Fig. 3). Interestingly, the signal of Re was detected over the whole cell, as it would be expected in the case of a compound bound to a membrane protein. However, the Re signal seemed to be higher at the nucleus. This could be explained by a labeling of carbonic anhydrase II, as recently published for another LDAI reagent.^48^ Indeed, this protein is evenly distributed in the cell, and a higher signal is expected at the nucleus where the cell is the thickest. Cells incubated with the LDAI-SCoMPI in the presence of EZA did not contain any detectable Re (Fig. S7†). This confirms that the Re signal observed previously was not due to a non-specific labeling but results from the labeling of the carbonic anhydrases IX, XII and/or CAII. Rhenium-based complexes are thus suitable for labeling of membrane bound proteins.

**Fig. 3:**
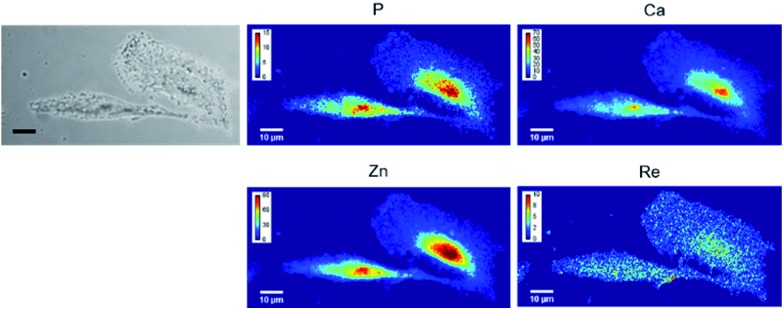
XRF imaging of Re-labeled endogenous carbonic anhydrases. A549 cells were grown on Si_3_N_4_ slides for 24 h at 37 °C. They were put under hypoxic conditions for 24 h, and then incubated for 3 h at 37 °C in the presence of 10 μM LDAI-SCoMPI under normoxic conditions. The images from the left show the bright field image of the measured cells, and the distribution of P, Ca, Zn, and Re measured by SXRF-MS. Measurement conditions: 13.5 keV, beam size: 300 × 300 nm^2^ and image pixel size: 300 nm. The XRF peak intensities were calculated using the PyMca software and are shown in counts per s.

## Conclusions

In conclusion, the results presented herein showed that it was possible to label and map by X-ray fluorescence an exogenous protein in CHO cells and membrane bound proteins in A549 cells, which establishes the high sensitivity of Re-based probes for X-ray imaging. This proof of concept offers new perspectives in XRF imaging, for which so far no specific graftable tags were available. This third modality of SCoMPI opens new opportunities for correlative imaging.

## Conflicts of interest

There are no conflicts to declare.

## Supplementary Material

Supplementary information
