# Supplementary material for: Graftable SCoMPIs enable the labeling and X-ray fluorescence imaging of proteins
Source: Chem Sci. 2018 Apr 13;9(19):4483–7. doi: 10.1039/c8sc00886h (PMC5958345; doi:10.1039/c8sc00886h)
Supplement: Supplementary file 1 [file SC-009-C8SC00886H-s001.pdf]

Electronic Supplementary Material (ESI) for Chemical Science.
This journal is © The Royal Society of Chemistry 2018

Supporting Information

Graftable SCoMPIs enable the labeling and X-Ray fluorescence imaging of proteins

Sarah Hostachy,[a] Marie Masuda,[b] Takayuki Miki,[b] Itaru Hamachi,[b] Sandrine Sagan,[c] Olivier Lequin,[c] Kadda Medjoubi,[d] Andrea Somogyi,[d] Nicolas Delsuc*[a] and Clotilde Policar*[a]

Contents

1 Supplementary figures and tables 2
2 Synthesis 13
2.1 General considerations 13
2.2 Synthesis of a thiol-reactive SCoMPI 13
2.3 Synthesis of LDAI-SCoMPI for the labeling of endogenous Carbonic Anhydrases 18
2.3.1 Synthesis of the SCoMPI-Acyl moiety 18
2.3.2 Synthesis of the ligand-imidazole moieties 22
2.3.3 Synthesis of the SCoMPI-LDAI 36
3 Protein production and purification 40
4 Protein labeling 41
4.1 *In vitro* labeling of Homeodomains 41
4.2 Labeling of Carbonic Anhydrases with LDAI-SCoMPI 41
4.2.1 Stock solutions 41
4.2.2 *In vitro* labeling of human Carbonic Anhydrase 1 (hCA1) 41
5 Imaging of SCoMPI-labeled proteins in cells 42
5.1 Stock solutions 42
5.2 Incubation conditions 42
5.2.1 SCoMPI-labeled homeodomains 42
5.2.2 SCoMPI-LDAI for the labeling of CA-IX and CA-XII 42
5.3 Synchrotron X-ray Fluorescence microspectroscopy 42
6 References 45

1 Supplementary figures and tables

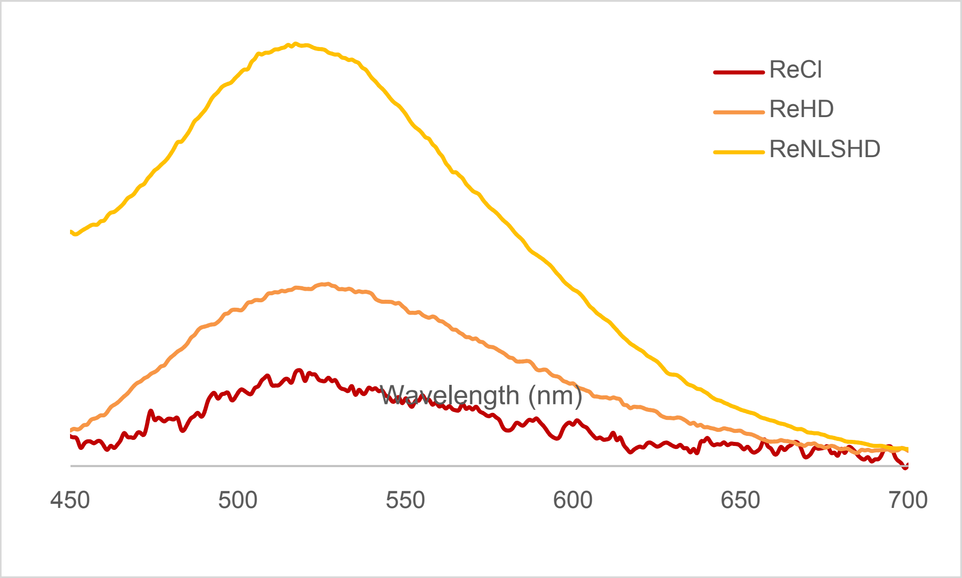

Figure S1 Luminescence properties of labeled homeodomains. Emission spectra of Re-HD (orange) Re-NLS-HD (yellow) and of a [Re(CO)$_3$(Cl)(Pyta)] complex. Emission spectra were recorded for excitation at 350 nm with 5 nm slits for excitation and emission, in PBS buffer (proteins) or H_2O+2% DMSO (ReCl complex).

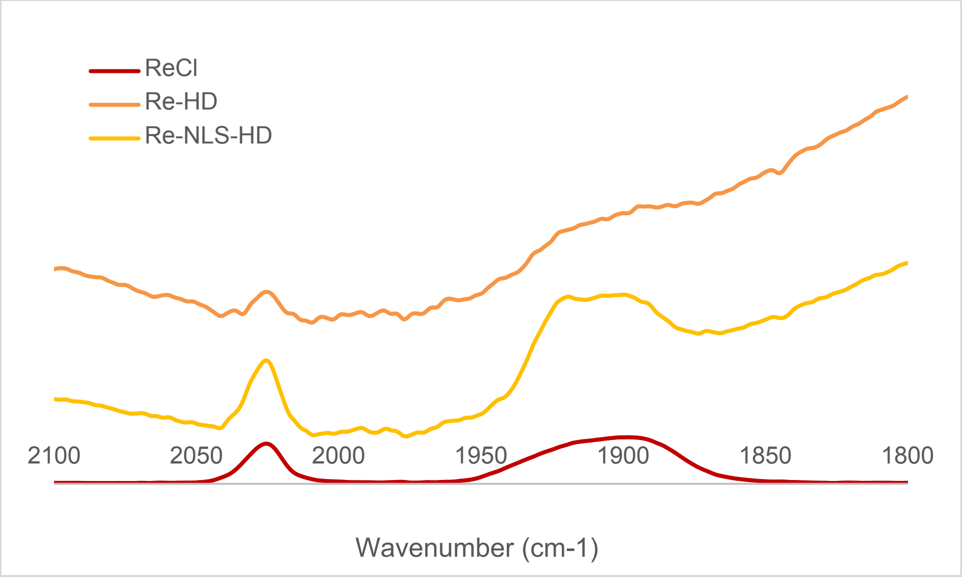

Figure S2 FTIR spectra of labeled homeodomains. FTIR spectra of Re-HD (orange), Re-NLS-HD (yellow), and a [Re(CO)$_3$(Cl)(Pyta)] complex.

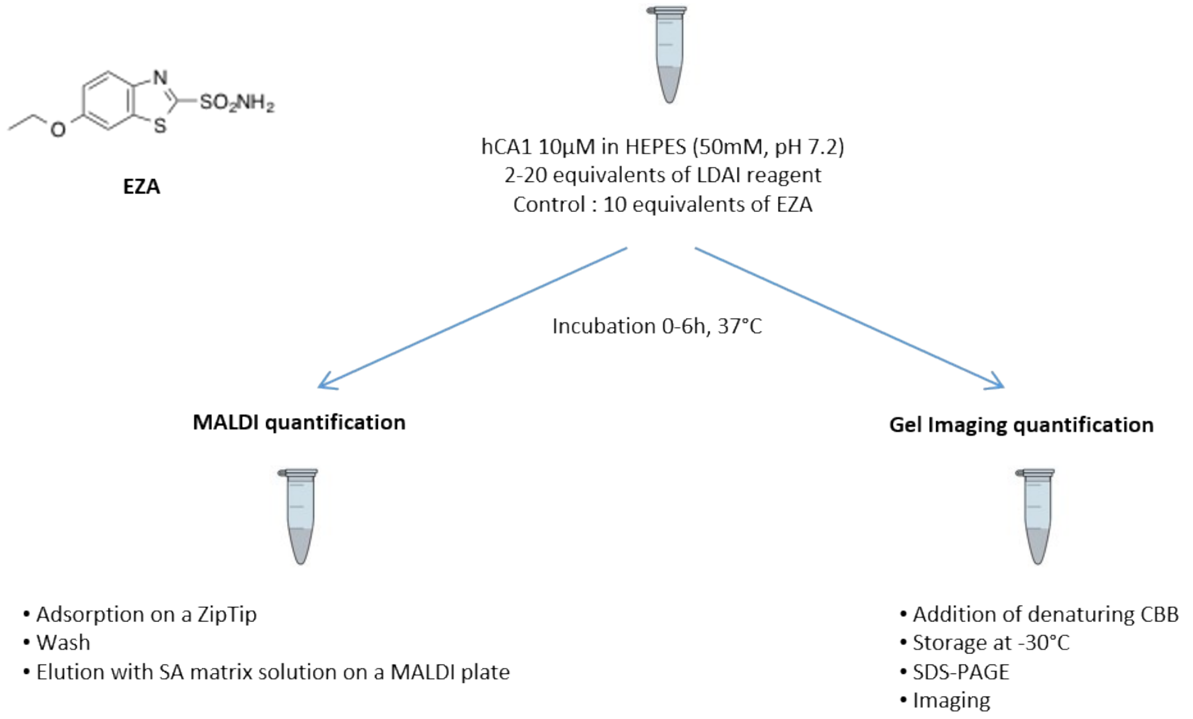

Figure S3 Workflow for the quantification of the labeling of hCA-I by MALDI-TOF MS and fluorescence gel imaging. *For MALDI-TOF MS quantification*, 10 µL aliquots of the incubation solution (10 µM hCA-I, 2-20 equiv. LDAI-SCoMPI, 0-10 equiv. EZA; 37 °C) were taken every two hours for 6 h, quenched by adsorption on C18 ZipTip® and washings with 0.1% aqueous TFA, and eluted/spotted on a MALDI plate with the matrix solution. MALDI spectra were then recorded and peak areas of labeled and unlabeled protein were measured to determine the labeling percentage. For fluorescence gel imaging, 30 µL aliquots of incubation solution were taken at given time points, diluted with 2X denaturing buffer and stored at −30°C until SDS-Page analysis. After gel migration, fluorescence imaging was performed prior to CBB staining.

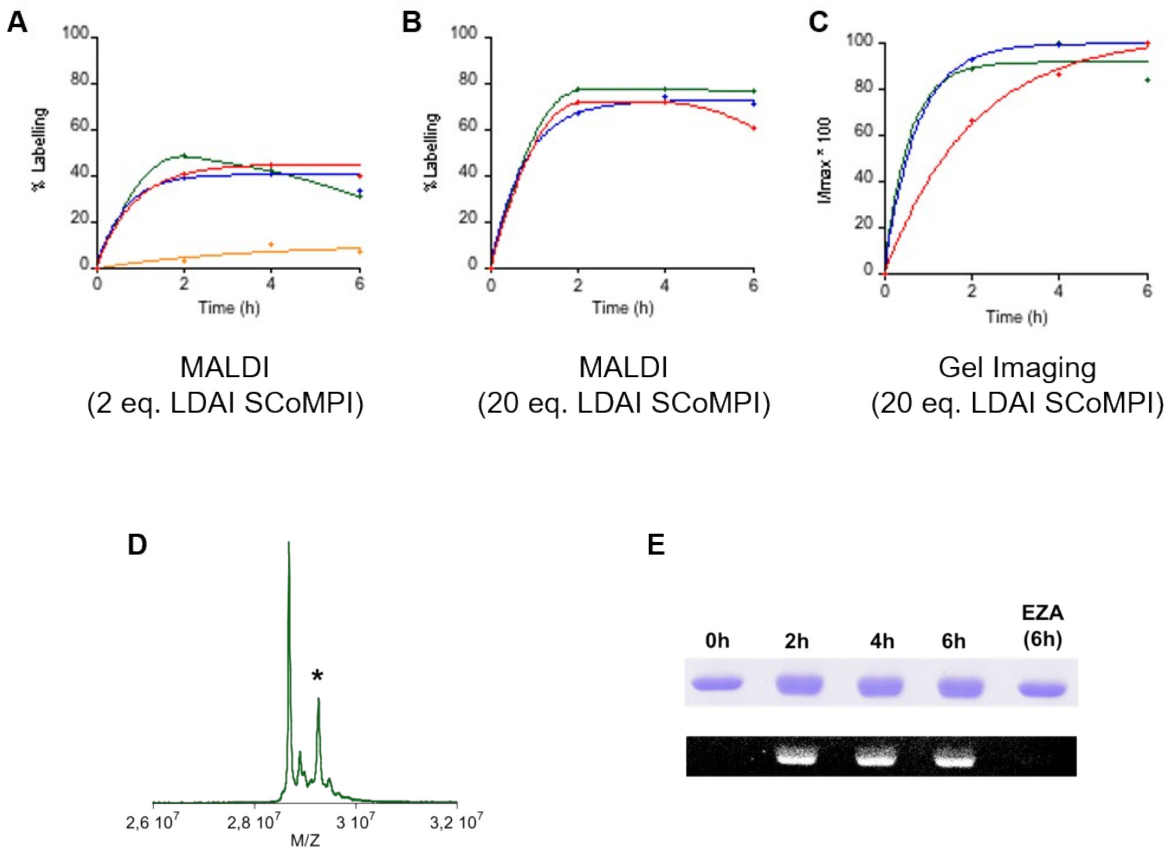

Figure S4 Quantification of the *in vitro* labeling of hCA-I by LDAI-SCoMPI reagents. (*A) MALDI-TOF MS:* Time profiles of labeling of hCA-I (10 µM in 50 mM HEPES buffer pH 7.2) for incubation at 37°C with 2 equivalents of compound 14a (orange, ReC0), 14b (blue, ReC3), 14c (red, ReC6), 14d (green, ReC5). % Labeling represents the ratio of the area of the labeled protein peak over areas of labeled and unlabeled proteins. *(B) MALDI-TOF MS*: Time profiles of labeling of hCA-I (10 µM in 50 mM HEPES buffer pH 7.2) for incubation at 37 °C with 20 equivalents of 14b (blue, ReC3), 14c (red, ReC6), 14d (green, ReC5); *(C) Fluorescence gel imaging:* Time profiles of labeling of hCA-I (10 µM in 50 mM HEPES buffer pH 7.2) for incubation at 37 °C with 20 equivalents of 14b (blue, ReC3), 14c (red, ReC6), 14d (green, ReC5). Area mean intensities were measured using ImageJ software, and the ratio of the sample intensity over maximal intensity plotted. (D) Example of MALDI-TOF spectrum of hCA-I(10 µM) incubated with 14d. The labeled protein peak is starred (*). *(E) Example of SDS-PAGE analysis for the labeling of hCA-I with 20 equivalents of 14d.* top: CBB stained, bottom: fluorescence imaging.

a)

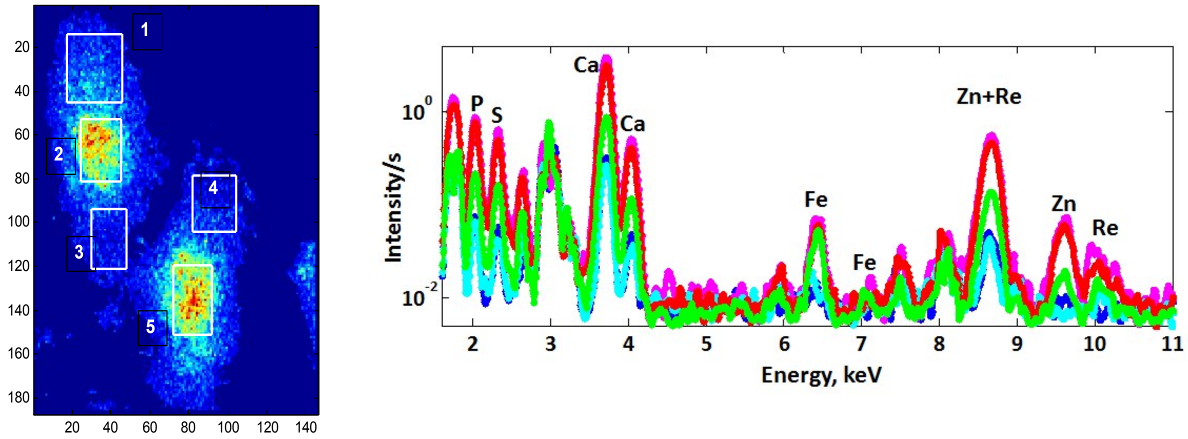

b)

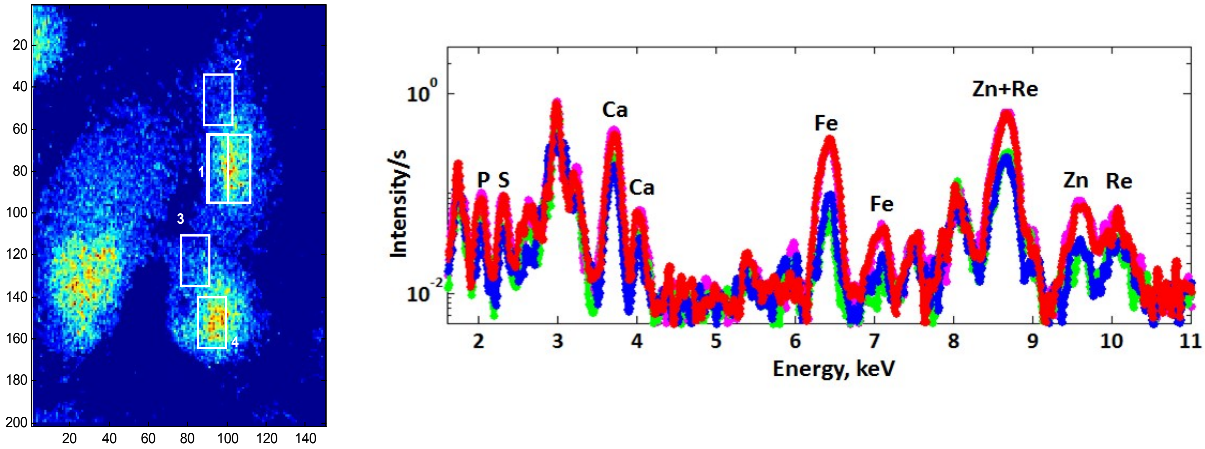

Figure S5 XRF Imaging of Re-labeled Engrailed homeodomains. Conditions: excitation energy 13.5 keV, beam size: 300 x 300 nm^2 pixel size: 300 nm, accumulation time: 4.0 s /pixel for Re-HD and 1.2 s/pixel for Re-NLS-HD. All the Re distribution maps and the average XRF spectra are normalized to 1s/pixel accumulation time. a) Re-HD: cell distribution of Zn showing regions of interest (ROIs) and corresponding XRF spectra (ROI1 green, ROI2 red, ROI3 blue, ROI4 cyan, ROI5 magenta). b) Re-NLS-HD: cell distribution of Zn showing regions of interest (ROIs) and corresponding XRF spectra (ROI1 red, ROI2 blue, ROI3 green, ROI4 magenta).

a)

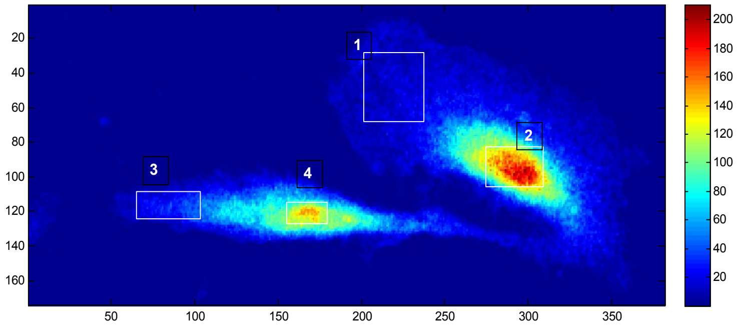

b)

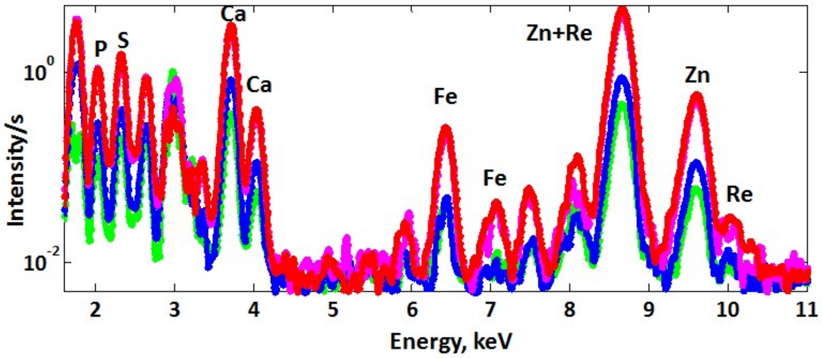

Figure S6 XRF Imaging of Re-labeled endogenous Carbonic Anhydrases. Conditions: excitation energy: 13.5 keV, pixel size: 300 x 300 nm^2, accumulation: 1.8 s /pixel. a) Cell distribution of Zn showing regions of interest (ROI). b) XRF spectra corresponding to the ROIs: ROI1 green, ROI2 red, ROI3 blue, ROI4 magenta. The Re distribution map and the average XRF ROI-spectra are normalized to 1s/pixel accumulation time.

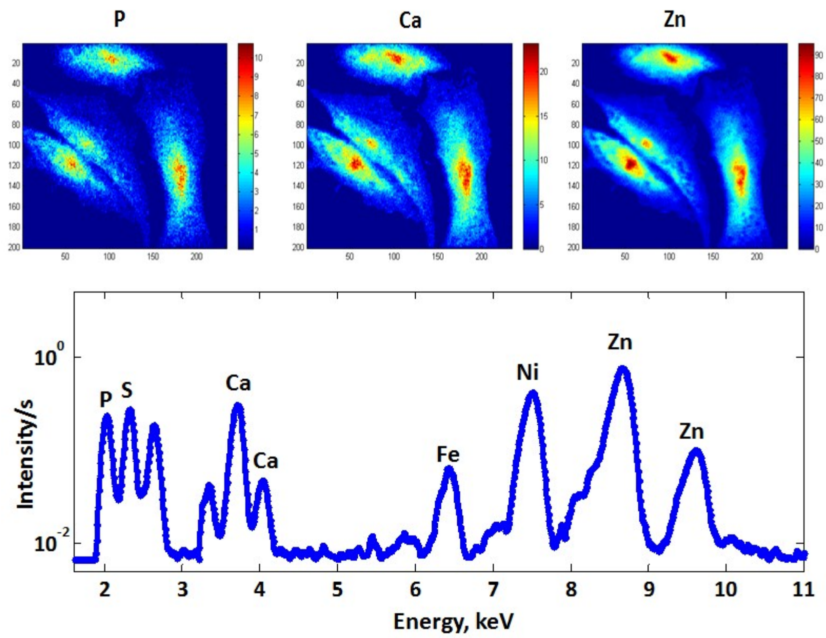

Figure S7 XRF sum spectrum of control experiment. Conditions: excitation energy: 13.5 keV, pixel size: 300 x 300 nm^2, accumulation time: 4.8 s /pixel. a) Cell distribution of P, Ca, and Zn b) average XRF pixel-spectrum calculated from the total XRF spectra of the 4 cells. The elemental distribution maps and the average XRF pixel-spectrum are normalized to 1s/pixel accumulation time**.**

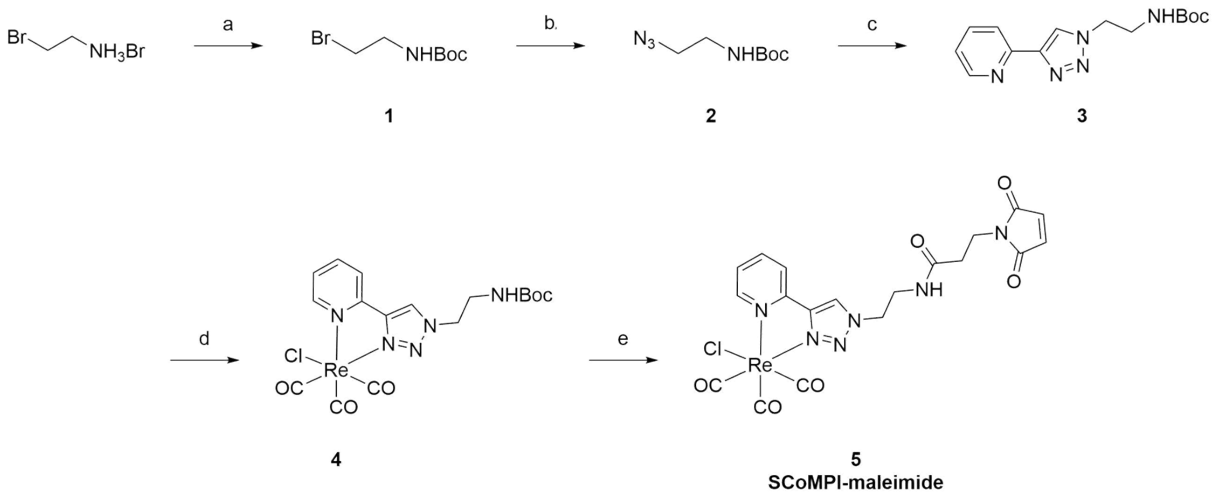

Scheme S1 Synthesis of a thiol-reactive SCoMPI for cystein labeling. Reaction conditions: (a) Boc_2O, DIEA, THF, 1 h, 0 °C to RT, 99%; (b) NaN_3, NaI, acetone:water (3:1 v:v), 48 h, RT, 77%; (c) 2-ethynylpyridine, $CuSO_4$, sodium ascorbate, acetone:water (2:1 v:v), 2h, RT, 78%; (d) $Re(CO)_5Cl$, toluene, 6h, 80 °C, 99%; (e) i. TFA:DCM (1:1 v:v), 1 h, RT, ii. 3-maleimidopropionic acid *N*-hydroxysuccinimide ester, DIEA, dry DMF, overnight, RT.

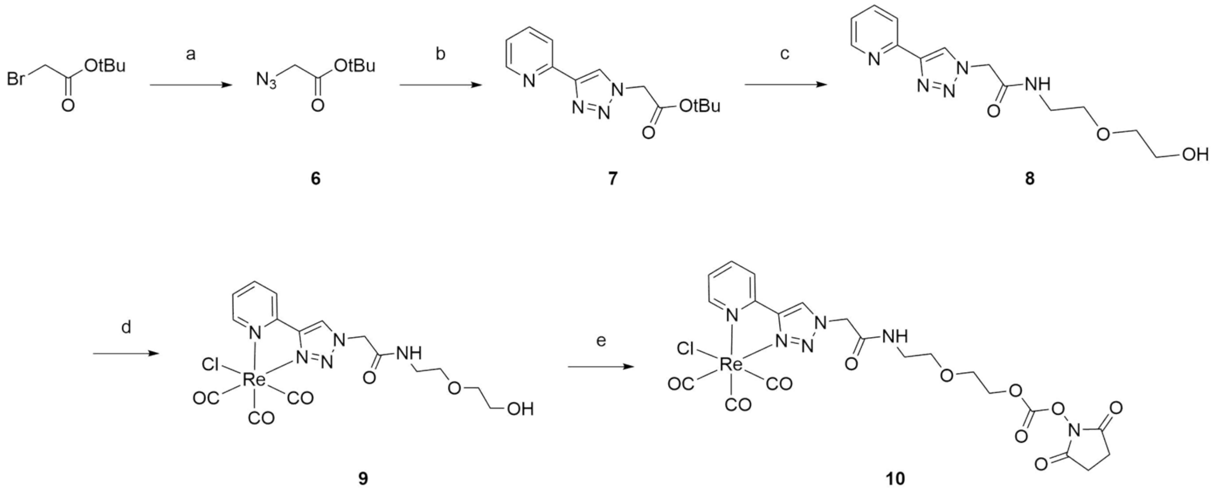

Scheme S2 Synthesis of the SCoMPI-Acyl moiety. Reaction conditions; (a) (a) NaN_3, acetone:water (3:2 v:v), reflux, 20 h, 97%; (b) 2-ethynylpyridine, $Cu(OAc)_2$, sodium ascorbate, *t*-BuOH : H_2O (1:1 v:v), 1 h 30, RT, 93%; (c) i. TFA:DCM 50:50, 2 h, RT, ii. EDC, 2-(2-aminoethoxy)-ethanol, DIEA, 40%; (d) $Re(CO)_5Cl$, MeOH, reflux, overnight, 99%; (e) *N,N'*-disuccinimidyl carbonate, triethylamine, dry DMF, 6 h, RT.

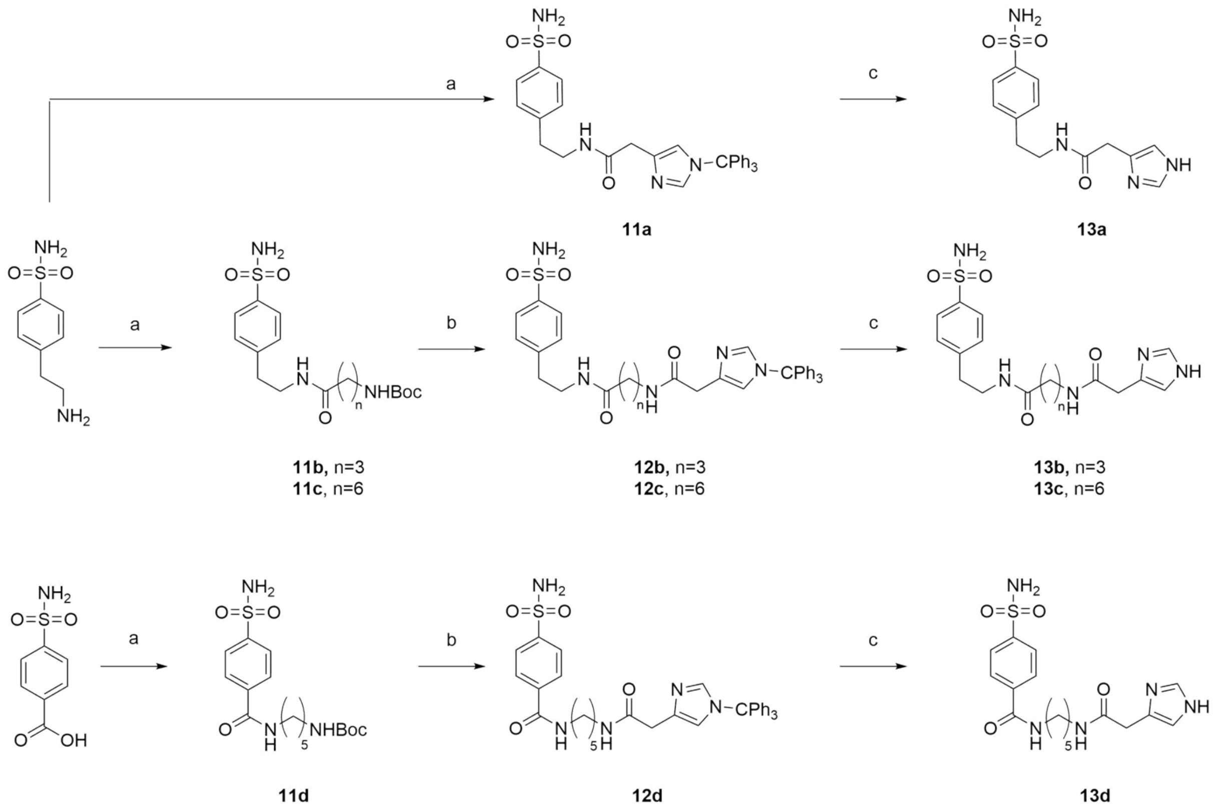

Scheme S3 Synthesis of the ligand-imidazole moieties. Reaction conditions: (a) EDC·HCl, HOBT·H_2O, DIEA, linker, overnight, RT, 40-74%; (b) *i.* TFA:DCM (1:1), 1h, RT; *ii.* EDC·HCl, HOBT·H_2O, DIEA, 1-(triphenylmethyl)-1H-imidazole-4-acetic acid, overnight, RT, 34-54%; (c) TIS, TFA:DCM (1:5), 1h, RT, 85-93 %.

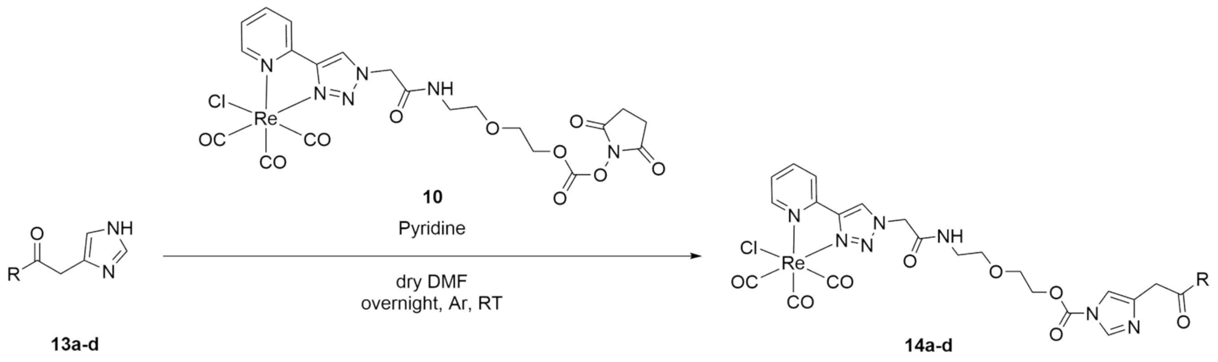

Scheme S4 Synthesis of the LDAI-SCoMPI.

**Lysis buffer**	- 20mM sodium phosphate buffer pH 7.5<br>- 500mM NaCl<br>- 1mM DTT<br>- 25 mM imidazole
**Elution buffer His-trap**	- 20mM sodium phosphate buffer pH 7.5<br>- 500mM NaCl<br>- 1mM DTT<br>- 500 mM imidazole
**PreScission cleavage buffer**	- 20mM sodium phosphate buffer pH 7.5<br>- 150mM NaCl<br>- 1mM DTT<br>- 1mM EDTA
**Thiol-maleimide reaction buffer**	- 50mM sodium phosphate buffer pH 6.5<br>- 150mM NaCl<br>- 10mM EDTA<br>- 1 equiv. de TCEP (ex : 0.4mM)

Table S1 Composition of buffers for protein purification. pH of the buffers was adjusted using concentrated NaOH or HCl solutions. DTT and TCEP were added to the buffers immediately prior to use. Buffers used for FPLC were filtered over 0.2 µm membrane.

**Cys-HD**	*GPCAS* --------------- -DKRPRTAFTA EQLQRLKAEF QTNRYLTEQR RQSLAQELGL NESQIKIWFQ NKRAKIKKAT *QA*
**Cys-NLS-HD**	*GPCAS* **SGPRSRKPKK KNPNKE**DKRP RTAFTAEQLQ RLKAEFQTNR YLTEQRRQSL AQELGLNESQ IKIWFQNKRA KIKKAT

Table S2 Sequence of Homeodomain constructs (NLS sequence in bold). HD and NLS-HD constructs correspond to residues 200-259 and 184-259 of chick Engrailed 2 homeoprotein, respectively. Extra residues that do not belong to the wild type sequence are in italics.

	**Cys-HD**	**Cys-NLS-HD**	**Re-HD**	**Re-NLS-HD**
MW (kDa)	7.821	9.455	8.465	10.101
M/z	[M]: 7816	[M]: 9449	[M]: 8462<br>[M-Cl]: 8427<br>[M-Re(CO)$_3$Cl] : 8156	[M]:10095<br>[M-Cl]:10060
ε^0_{280} ($M^{-1}.cm^{-1}$)	7.0 × 10^3	7.0 × 10^3	16.9 × 10^3	16.9 × 10^3

Table S3 Some properties of labeled and unlabeled homeodomain constructs. MW and extinction coefficients of unlabeled proteins were calculated using the ProtParam tool of ExPASy Resource Portal (http://www.expasy.org/).

2 Synthesis

2.1 General considerations

Chemicals and solvents

All chemicals and solvents were purchased from commercial sources (Sigma-Aldrich, Acros, Alfa-Aesar, TCI, Strem, Iris) and were used as received. Dry solvents were purchased from Acros and Sigma and used as received.

Purification

Preparative flash chromatography was performed using Normasil 60 (40–60 µm) silica or on a Reveleris® purification system using prepacked cartridges. Analytical HPLC measurements were run on a Dionex Ultimate 3000 instrument using C18 ACE® column (250 × 4.5 mm) packed with spherical 5 µm particles of 300 Å pore size at $1 mL\cdot min^{-1}$. Preparative HPLC consisted of a dual wavelength UV-Vis absorbance detector (Waters 2487) and a Waters 600 preparative pump. Purification of crude products was achieved with a C18 Nucleodur® preparative column ((250×16 mm) packed with spherical 5 µm particles of 300 Å). Experiments were carried out at a flow rate of 14mL · min−1 at room temperature.

Characterization

^{1}H and ^{13}C NMR spectra were recorded on a Bruker DRX300 or a Bruker AM-400 MHz spectrometer using solvent residual peaks as internal standards.[1] When $CDCl_3$:CD_3OD is indicated as a solvent, a 1:1 ratio of $CDCl_3$:CD_3OD was used as NMR solvent. In this case, the chemical shifts were calibrated using the residual peak of methanol. J values are given in Hertz.

High resolution mass spectra (HR-MS) were obtained on a Bruker hybride APEX spectrometer (ESI). Proteins were characterized by MALDI-TOF-MS in the positive ion reflector mode on an ABI Voyager DE-Pro MALDI-TOF mass spectrometer (Applied Biosystems) using as matrix a saturated solution of CHCA (α-cyano-4-hydroxycinnamic matrix) or SA (sinapinic acid) in ACN/H_2O/TFA (50:50:0.1 v:v:v).

UV-visible absorption spectra were recorded on a Varian Cary 300 Bio spectrophotometer, luminescence emission spectra on a Jasco FP-8300 spectrofluorimeter, IR spectra on a Perkin-Elmer Spectrum 100 FT-IR spectrometer.

2.2 Synthesis of a thiol-reactive SCoMPI

*N-(tert-butoxycarbonyl)-2-bromoethylamine (**1**)*

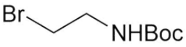

2-bromoethylamine hydrobromide salt (1.02 g, 4.98 mmol, 1 equiv.) was suspended in THF (80 mL). Di-*tert*-butyl dicarbonate (1.23g, 5.64 mmol, 1.1 equiv.) was then added. The white suspension was cooled in an ice bath, and DIEA (1.6 mL, 9.95 mmol, 2 equiv.) was added dropwise. The reaction mixture was stirred for 1 hour, and solvent was removed by rotary evaporation. The resulting crude was dissolved in EtOAc and washed once with 10% aqueous $NaHCO_3$ and once with brine. The organic layer was then dried over anhydrous $MgSO_4$, filtered and concentrated to give the desired compound as a colorless oil (1.107 g, 4.94 mmol, 99%).

^{1}H-NMR (300 MHz, $CDCl_3$): δ (ppm) 4.95 (s, 1H), 3.54-3.51 (m, 2H), 3.47-3.44 (m, 2H), 1.45 (s, 9H); in agreement with the literature.[2]

*N-(2-azidoethyl)-N-(tert-butoxycarbonyl)amine (**2**)*

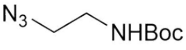

N-(*tert*-butoxycarbonyl)-2-bromoethylamine (1) (616.8 mg, 2.75 mmol, 1 equiv.) was dissolved in a mixture of acetone and water (10.7 mL, acetone:H_2O 3:1 v:v). The solution was warmed to 45 °C (bath

temperature), then sodium azide (359.2 mg, 5.52 mmol, 2 equiv.) and sodium iodide (354.0 mg, 2.36 mmol, 0.85 equiv.) were added. The mixture was stirred overnight, then more sodium azide (187.4 mg, 2.88 mmol, 1 equiv.) was added and the reaction mixture was stirred for 5 more hours. DCM (15 mL) and H_2O (5 mL) were added to reaction mixture, and the mixture was decanted. The aqueous layer was extracted once more with DCM (5 mL). Organic layers were pooled, dried over anhydrous $MgSO_4$, filtered and concentrated to give *N*-(*tert*-butoxycarbonyl)-2-azidoethylamine as a colorless oil (394.0 mg, 2.11 mmol, 77%).

<u>^{1}H-NMR (300 MHz, $CDCl_3$)</u>: δ (ppm) 4.84-4.80 (br, 1H, N*H*), 3.41 (t, J = 5.4, 2H), 3.30 (t, J = 5.6, 2H), 1.44 (s, 9H).

<u>^{13}C-NMR (75 MHz, $CDCl_3$)</u>: δ (ppm) 155.83, 79.78, 51.25, 40.10, 28.40 (3C).

NMR data are in agreement with the literature.[3]

*PytaNHBoc (**3**)*

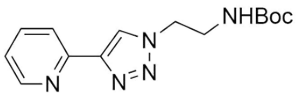

N-(2-azidoethyl)-*N*-(*tert*-butoxycarbonyl)amine (**2**) (568 mg, 3.05 mmol, 1 equiv.) was dissolved in a mixture of acetone and water (30 mL acetone:water 2:1 v:v). Anhydrous copper sulfate (131 mg, 0.82 mmol, 0.25 equiv.) and sodium ascorbate (153.8 mg, 0.77 mmol, 0.25 equiv.), followed by 2-ethynylpyridine (310 µL, 3.07 mmol, 1 equiv.), were added. The mixture was sonicated for a few minutes, and reaction mixture was stirred at room temperature for 2 hours. A light brown precipitate formed. Acetone was removed by rotary evaporation, and the aqueous layer was diluted with 28% aqueous ammonia solution. The aqueous layer was extracted once with DCM. The organic layer was then washed once more with 28% ammonia solution, and once with brine. It was then dried over anhydrous Na_2SO_4, filtered and concentrated. The resulting brown solid was purified by automated silica gel flash column chromatography (0-100% EtOAc in cyclohexane) to yield the desired compound as a white solid (692 mg, 2.39 mmol, 78%).

<u>^{1}H-NMR (300 MHz, $CDCl_3$)</u>: δ (ppm) 8.33 (d, J = 4.6, 1H), 8.01 (s, 1H), 7.90 (d, J = 7.9, 1H), 7.57 (td, J = 7.7, 1.7, 1H), 7.03 (ddd, J = 7.5, 4.9, 0.9, 1H), 5.99 (br, 1H), 4.41 (t, J = 5.4, 2H), 3.56 (q, J = 5.4, 2H), 1.30 (s, 9H).

<u>^{13}C-NMR (75 MHz, $CDCl_3$)</u>: δ (ppm) 155.91, 149.74, 149.01, 147.79, 136.60, 122.74, 122.53, 119.82, 79.41, 50.11, 40.57, 28.19 (3C).

<u>HR-MS (ESI+)</u>: m/z calculated for $[C_{14}H_{19}N_5O_2 + H]^+$: 290.16115, found: 290.16167, error: 1.8 ppm.

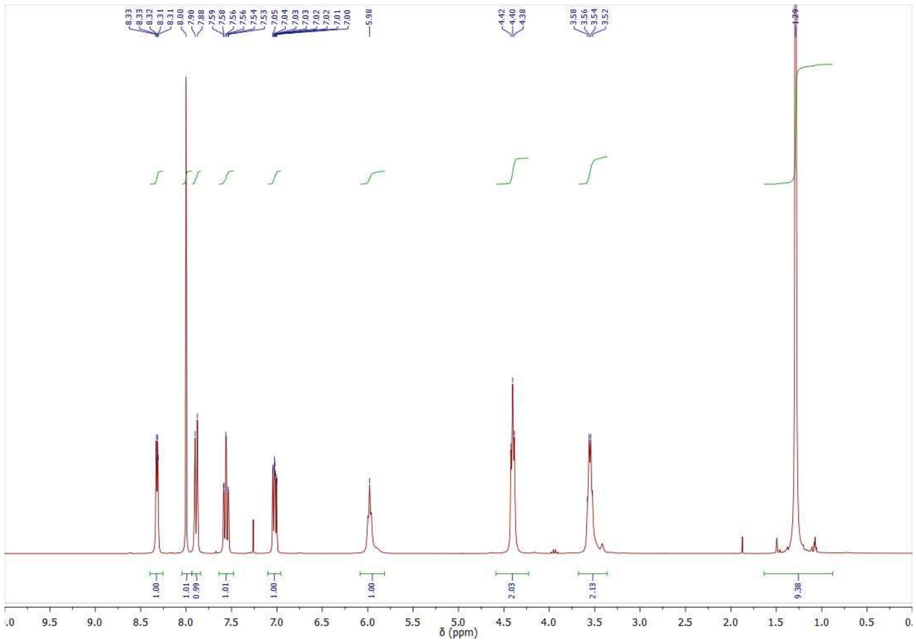

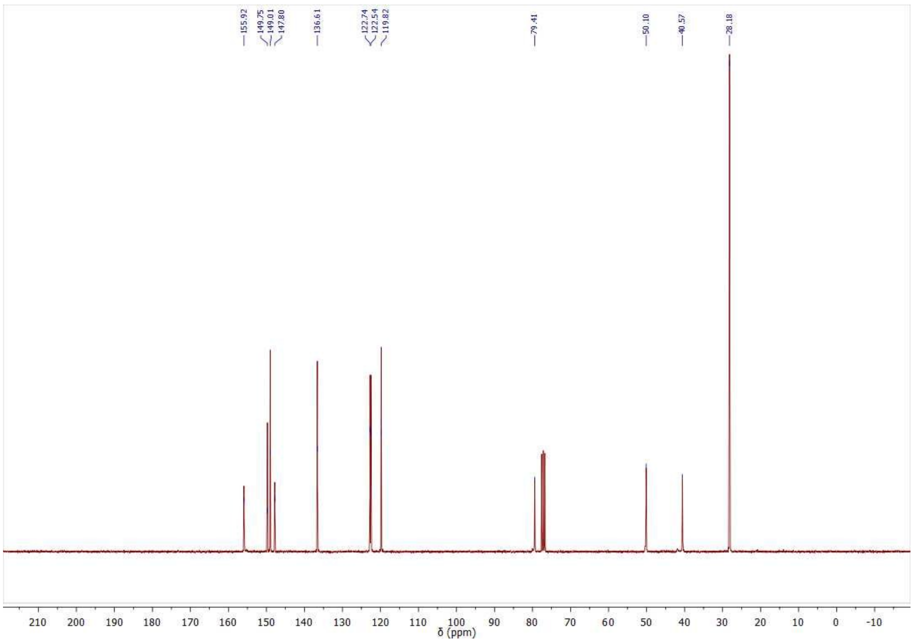

*[Re(CO)$_3$(Cl)(PytaNHBoc)] (**4**)*

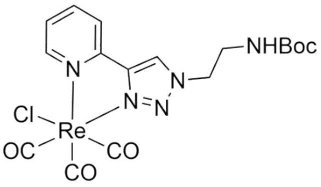

The ligand **3** (49.5 mg, 0.171 mmol, 1 equiv.) was dissolved in warm toluene (5 mL). Rhenium pentacarbonyl chloride (62.9 mg, 0.174 mmol, 1 equiv.) was added, and the mixture was heated at 80 °C (bath temperature) for 6 hours, during which a yellow precipitate formed. The yellow precipitate was filtered and washed with cold toluene to obtain the desired compound (99.7 mg, 0.168 mmol, 98%).

^{1}H-NMR (300 MHz, $CDCl_3$: CD_3OD): δ (ppm) 8.95 (d, J = 5.5, 1H), 8.77 (s, 1H), 8.10-8.02 (m, 2H), 7.48 (ddd, J = 6.8, 5.6, 2.1, 1H), 4.64-4.60 (m, 2H), 3.62 (t, J = 5.8, 2H), 1.38 (s, 9H).

^{13}C-NMR (75 MHz, $CDCl_3$: CD_3OD): δ (ppm) 197.3, 195.8, 188.8, 157.4, 153.7, 150.0, 149.4, 140.4, 126.5, 125.6, 122.9, 80.5, 52.4, 40.5, 28.5 (3C).

IR: ν_{ReCO}/cm^{-1} 2025, 1902.

HR-MS (ESI+): m/z calculated for $[C_{17}H_{19}ClN_5O_5Re + Na]^+$: 616.04965, found: 616.05007, error: 0.7 ppm.

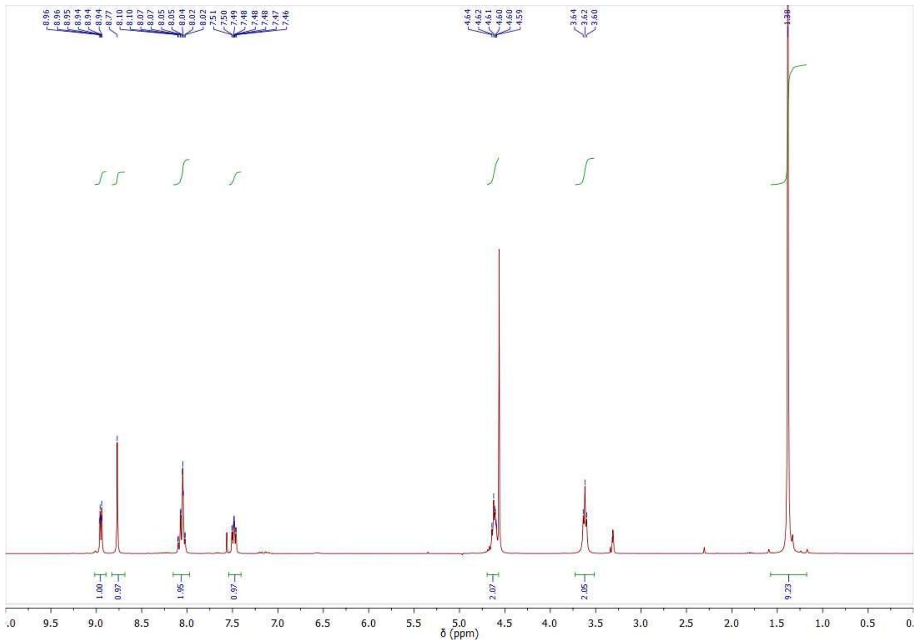

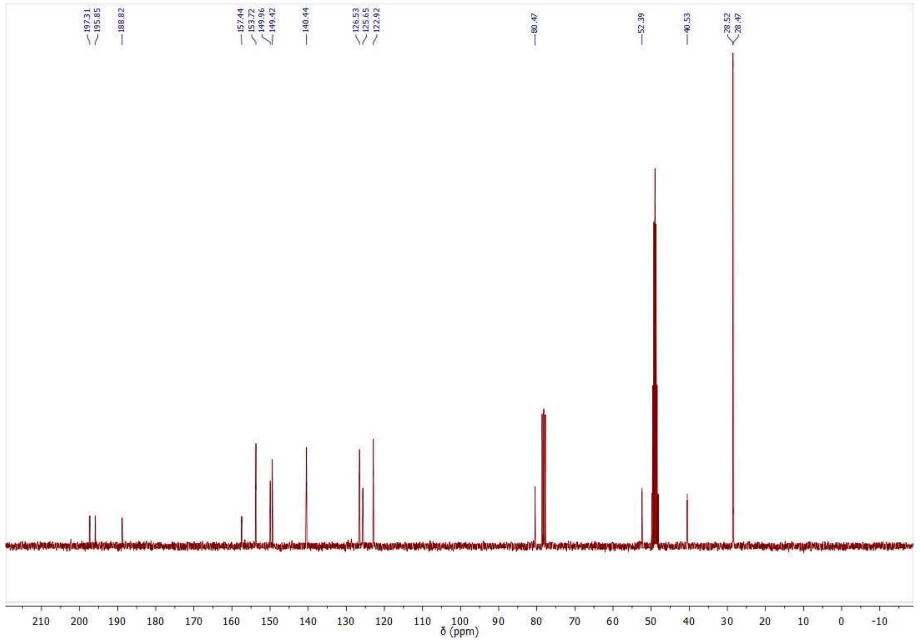

[Re(CO)$_3$(Cl)(PytaNH−maleimide)] (***5, SCoMPI-maleimide***)

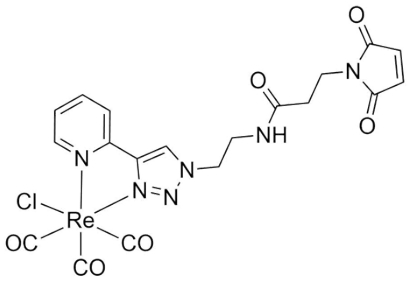

[Re(CO)$_3$(Cl)(PytaNHBoc)] derivative (30.5 mg, 51.3 µmol, 1.3 equiv.) was dissolved in DCM (1 mL) and TFA (1mL) was added drop wise. The reaction mixture was stirred at room temperature for 1 hour, then precipitated in cold Et$_2$O. The resulting solid was dried under vacuum, put under argon, and dissolved in dry DMF (1.2 mL). 3-maleimidopropionic acid N -hydroxysuccinimide ester (10.6 mg, 39.8 µmol, 1 equiv.), then DIEA (27 µL, 156 µmol, 3.9 equiv.), were added. Reaction mixture was stirred overnight at room temperature. DMF was removed by rotary evaporation and the resulting crude was

directly purified by HPLC (30 to 100 % ACN 0.1% TFA in 30 min, 14 mL/min, 9.3 min, purity: > 98%). The collected fraction was frozen in liquid nitrogen immediately after purification to prevent exchange of the chloride ligand.

<u>^{1}H-NMR (400 MHz, $CDCl_3$: CD_3OD)</u>: δ (ppm) 8.96 (ddd, J = 5.6, 1.4, 0.9, 1H, H^a pyta), 8.83 (s, 1H, H^g pyta), 8.10-8.08 (m, 1H, H^c pyta), 8.07 (dd, J = 1.7, 0.9, 1H, H^d pyta), 7.51 (ddd, J = 7.3, 5.6, 1.7, 1H, H^b pyta), 6.72 (s, 2H, *H* maleimide), 4.64 (t, J = 5.7, 2H, PytaCH_2), 3.76-3.72 (m, 4H, CH_2NHC(O)CH_2 and CH_2 α to maleimide), 2.42 (td, J = 6.8, 1.0, 2H, CH_2NHC(O)CH_2).

<u>^{13}C-NMR (101 MHz, $CDCl_3$: CD_3OD)</u>: δ (ppm) 197.4 (Re*C*O), 195.9 (Re*C*O), 189.3 (Re*C*O), 172.9 (CH_2*C*(O)NHCH_2), 171.4 (2C, *C*=O of maleimide ring), 153.7 (C^a pyta), 150.0 (C^e pyta), 149.5 (C^f pyta), 140.5 (C^c pyta), 134.9 (2C, *C*H of maleimide ring), 126.6 (C^g pyta), 126.0 (C^g pyta), 122.9 (C^d pyta), 51.7 (PytaCH_2), 39.5 (CH_2 α to maleimide), 35.17 (CH_2NHC(O)CH_2), 34.99 (CH_2NHC(O)CH_2).

<u>HR-MS (ESI+)</u>: m/z calculated for $[C_{19}H_{16}ClN_6O_6Re + Na]^+$: 667.02416, found: 667.02445, error: 0.4 ppm.

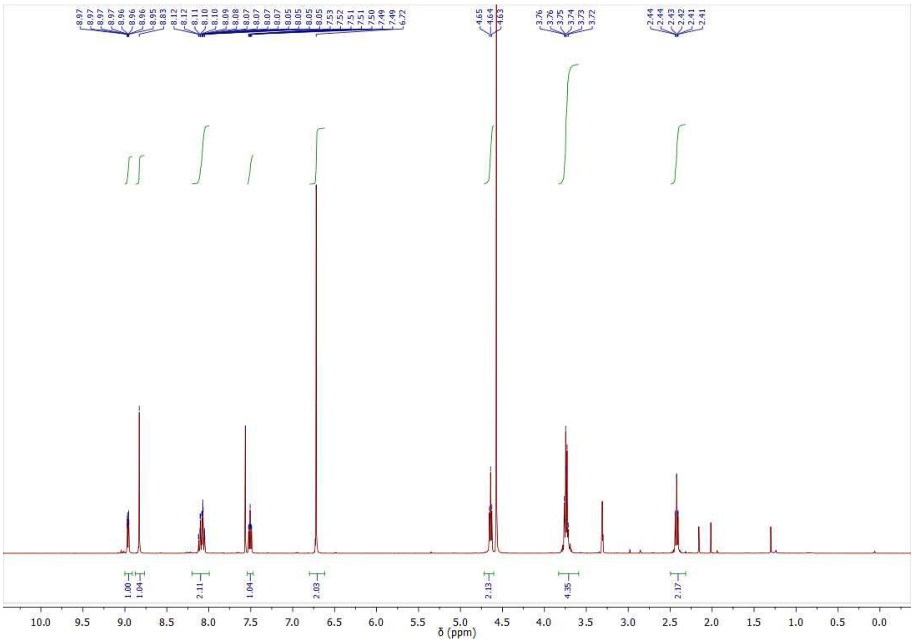

2.3 Synthesis of LDAI-SCoMPI for the labeling of endogenous Carbonic Anhydrases

2.3.1 Synthesis of the SCoMPI-Acyl moiety

*2-azidoacetic acid, tert-butyl ester (**6**)*

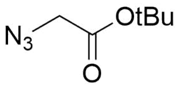

2-bromoacetic acid tert-butyl ester (1 mL, 6.8 mmol, 1 equiv.) was diluted in a 3:2 (v:v) mixture of acetone (4.1 mL) and water (2.7 mL). Sodium azide (664 mg, 10.2 mmol, 1.5 equiv.) was added, and the mixture was refluxed (sand bath temperature = 78 °C) for 20 h. The solution was cooled to room temperature and acetone was removed by rotary evaporation. The solution was diluted with water (6 mL) and extracted three times with Et_2O (3 × 6 mL). The organic layers were pooled, dried over anhydrous $MgSO_4$, filtered and concentrated to the desired compound as a colorless oil (1.034 g, 6.58 mmol, 97%).

<u>^{1}H-NMR (300 MHz, $CDCl_3$):</u> δ (ppm) 3.70 (s, 2H), 1.45 (s, 9H).

<u>^{13}C-NMR (75 MHz, $CDCl_3$):</u> δ (ppm) 167.45, 83.06, 50.97, 28.07.

NMR data in agreement with literature.[4,5]

*2-(1-[acetic acid tert-butyl ester]-1H-1,2,3-triazol-4-yl)pyridine (**7**, PytaCOOtBu)*

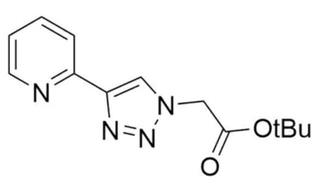

Compound **7** was synthesized by adapting published procedure from Benoist *et al*.[4]

2-azidoacetic acid *tert*-butyl ester (902.1 mg, 5.74 mmol, 1 equiv.) was dissolved in a1:1 mixture of *t*BuOH (28 mL) and water (28 mL). Copper acetate (278 mg, 1.40 mmol, 0.25 equiv.), sodium ascorbate (276.8 mg, 1.40 mmol, 0.25 equiv.) and 2-ethynylpyridine (560 µL, 5.54 mmol, 0.96 equiv.) were successively added. The mixture was sonicated for a few tens of seconds, during which a pale brown precipitate formed. The mixture was then stirred at room temperature until the solution turned green (1h30). The solution was diluted with DCM (60 mL) and extracted once. The organic layer was washed twice with aqueous saturated (28%) ammonia solution (2 × 60 mL) and once with brine (60 mL). The aqueous layers were extracted once more with DCM (60 mL), then both organic layers were pooled, dried over anhydrous Na_2SO_4, filtered and concentrated to the desired compound as a white solid (1.3821 g, 5.31 mmol, 93%).

<u>^{1}H-NMR (300 MHz, $CDCl_3$):</u> δ (ppm) 8.42 (d, J = 4.7, 1H), 8.18 (s, 1H), 8.00 (d, J = 7.9, 1H), 7.60 (td, J = 7.7, 1.4, 1H), 7.06 (dd, J = 6.8, 5.3, 1H), 5.02 (s, 2H), 1.31 (s, 9H).

<u>^{13}C-NMR (75 MHz, $CDCl_3$):</u> δ (ppm) 164.99, 149.90, 149.19, 148.37, 136.58, 123.47, 122.63, 119.93, 83.43, 51.43, 27.67 (3C).

*Pyta-OH (**8**)*

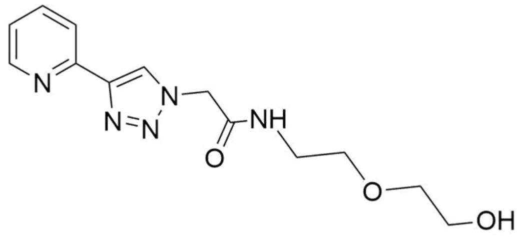

Compound **7** (1.597 g, 6.1 mmol, 1 equiv.) was dissolved in DCM (20 mL), and TFA (20 mL) was added slowly to the solution. The mixture was stirred at room temperature for 2h. Solvents were co-evaporated three times with toluene (3 × 30 mL). EDC· HCl (1.61 g, 8.4 mmol, 1.4 equiv.) was added to the resulting solid, and both solids were dissolved in dry DMF (30 mL) under argon. After 5-10 min stirring, 2-(2-aminoethoxy)ethanol (0.86 mL, 8.6 mmol, 1.4 equiv.) then DIEA (3.2 mL, 18.5 mmol, 3 equiv.) were added. Reaction mixture was stirred overnight at room temperature, and solvent was removed. The resulting crude was purified by flash silica gel column chromatography (0 to 10% MeOH in DCM) to yield the desired compound as a white solid (721 mg, 2.5 mmol, 40%).

<u>^{1}H-NMR (300 MHz, $CDCl_3$: CD_3OD):</u> δ (ppm) 8.29 (ddd, J = 5.0, 1.8, 0.9, 1H), 8.16 (s, 1H), 7.86 (dt, J = 8.0, 1.1, 1H), 7.61 (td, J = 7.8, 1.8, 1H), 7.07 (ddd, J = 7.6, 5.0, 1.2, 1H), 4.93 (s, 2H), 3.48-3.45 (m, 2H), 3.34-3.29 (m, 4H), 3.23-3.20 (m, 2H).

<u>^{13}C-NMR (75 MHz, $CDCl_3$: CD_3OD):</u> δ (ppm) 165.46, 149.23, 148.74, 147.28, 137.39, 123.92, 123.02, 120.31, 71.88, 68.81, 60.77, 52.00, 39.27.

<u>HR-MS (ESI+):</u> m/z calculated for $[C_{13}H_{17}ClN_5O_3 + Na]^+$: 314.12236, found: 314.12230, error: -0.2 ppm.

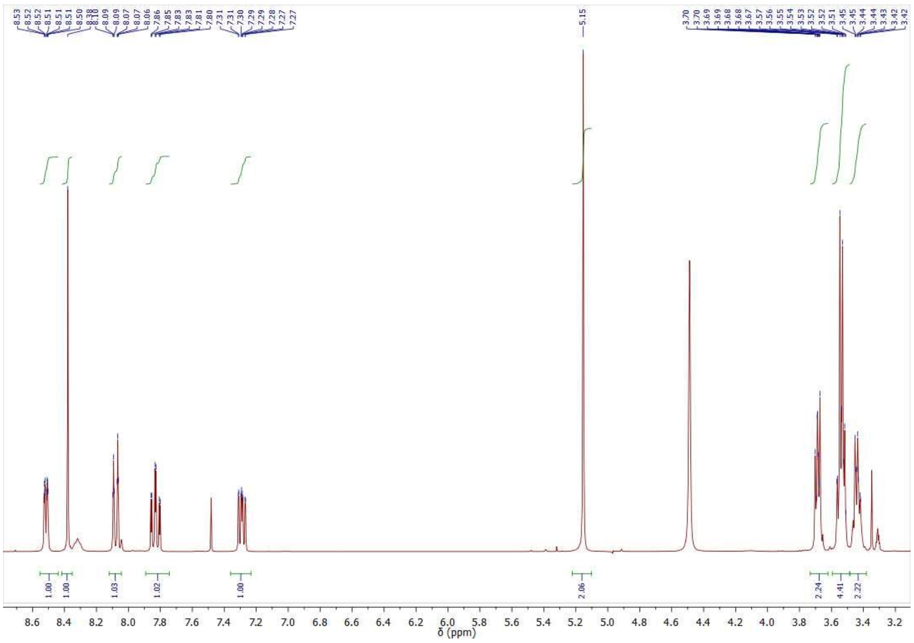

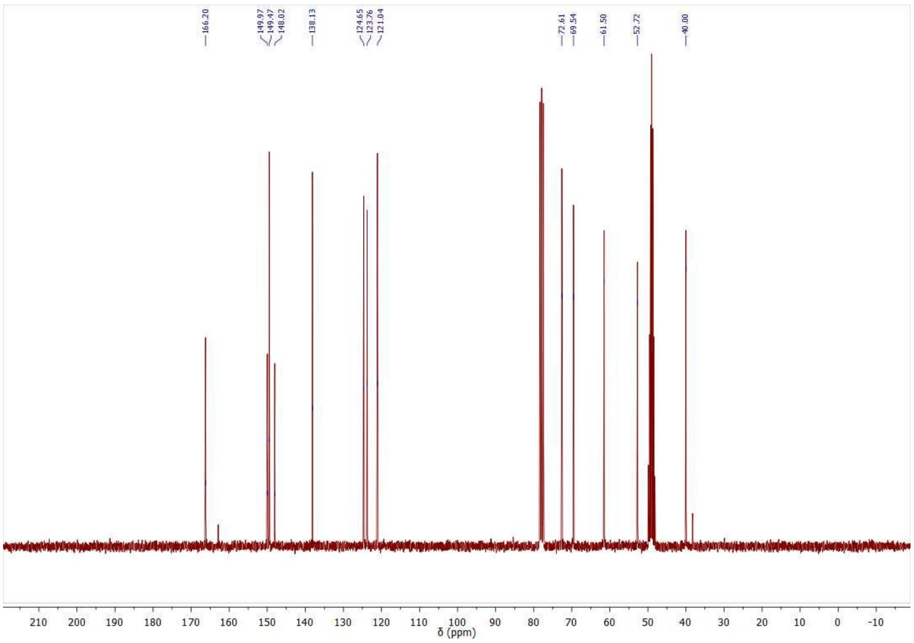

[Re(CO)$_3$(Cl)(Pyta-OH)] (***9***)

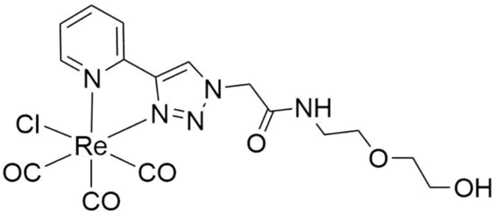

Ligand **8** (105.3 mg, 0.361 mmol, 1 equiv.) was dissolved in warm MeOH (1 mL). Re(CO)$_5$Cl (133.1 mg, 0.368 mmol, 1 equiv.) was added, and the mixture was refluxed overnight. The yellow precipitate that formed during this time was filtered and dried to give the desired complex (214.2 mg, 0.359 mmol, 99%).

<u>^{1}H-NMR (400 MHz, MeOH : CDCl$_3$):</u> δ (ppm) 8.96 (ddd, J = 5.6, 1.4, 1.0, 1H), 8.83 (s, 1H), 8.11-8.05 (m, 2H), 7.50 (ddd, J = 7.0, 5.6, 2.0, 1H), 5.28 (q, J = 20.7, 2H), 3.70 (t, J = 4.6, 2H), 3.60-3.56 (m, 4H), 3.48-3.45 (m, 2H).

<u>^{13}C-NMR (101 MHz, MeOH : CDCl$_3$):</u> δ (ppm) 197.5, 195.9, 189.3, 165.3, 153.6, 150.1, 149.6, 140.5, 126.76, 126.58, 123.0, 72.8, 69.7, 61.7, 53.8, 40.3.

<u>IR:</u> ν_{ReCO}/cm^{-1} 2019, 1891.

<u>HR-MS (ESI+):</u> m/z calculated for [$C_{16}H_{17}ClN_5O_6Re$ + Na]$^+$: 618.02918, found:618.02891, error: 0.4 ppm.

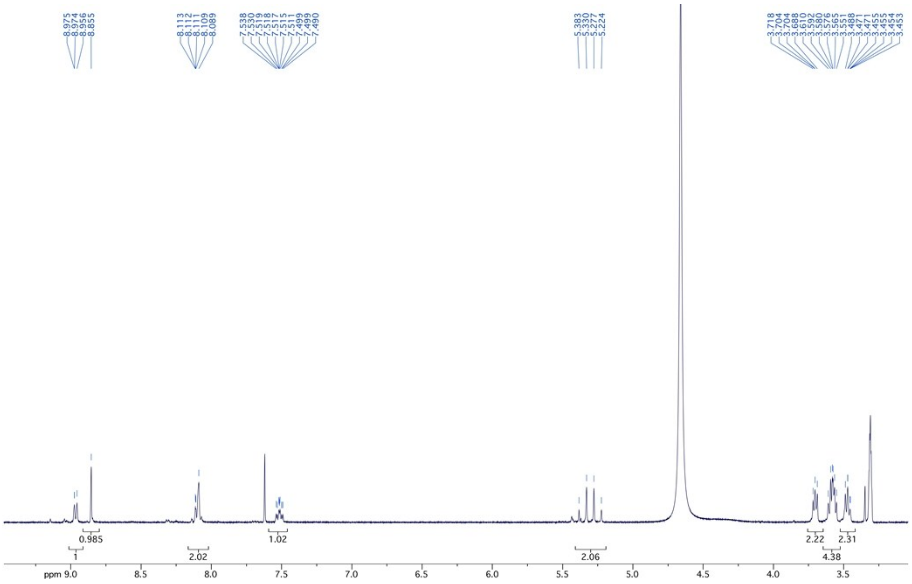

[Re(CO)$_3$(Cl)(Pyta-ONHS)] (***10***)

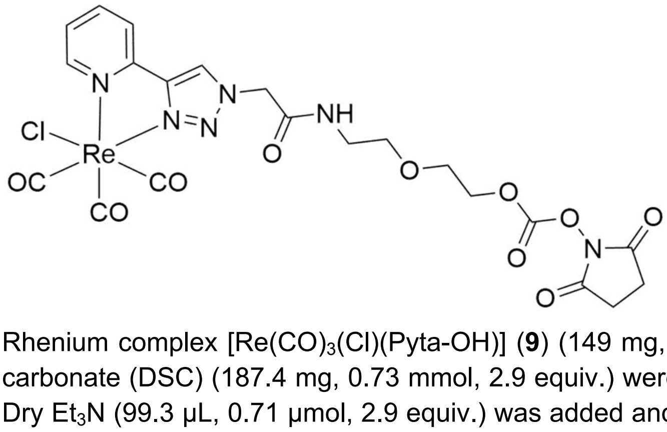

Rhenium complex [Re(CO)$_3$(Cl)(Pyta-OH)] (**9**) (149 mg, 0.25 mmol, 1 equiv.) and *N,N'*-disuccinimidyl carbonate (DSC) (187.4 mg, 0.73 mmol, 2.9 equiv.) were dissolved in dry DMF (2.4 mL) under Argon. Dry Et$_3$N (99.3 μL, 0.71 μmol, 2.9 equiv.) was added and the mixture was stirred at room temperature. After 2h30, more DSC was added (121.8 mg, 0.475 mmol, 1.9 equiv.) and the mixture was stirred at

50 °C (bath temperature) for another 3h30. DMF was removed by rotary evaporation. The resulting crude was dissolved in EtOAc and washed once with a saturated aqueous solution of NaHCO3. The organic layer was dried over anhydrous Na_2SO_4, filtered and concentrated. It was then purified by silica gel column chromatography (0 to 3 to 5 to 10% MeOH in DCM) to yield compound **10** as a yellow solid containing traces of impurities (106 mg).

<u>^{1}H-NMR (300 MHz, $CDCl_3$):</u> δ (ppm) 8.96 (d, J = 5.3, 1H), 8.60 (s, 1H), 8.04-7.95 (m, 2H), 7.41 (ddd, J = 7.1, 5.6, 1.7, 1H), 5.14 (q, J = 16.9, 2H), 4.48 (tdt, J = 13.0, 8.9, 4.4, 2H), 3.73 (t, J = 4.3, 2H), 3.58 (t, J = 4.6, 2H), 3.50-3.45 (m, 2H), 2.88 (s, 4H).

<u>^{13}C-NMR (75 MHz, $CDCl_3$):</u> δ (ppm) 197.1, 196.3, 188.9, 169.7 (2C), 164.2, 153.1, 151.7, 149.4, 148.8, 139.8, 126.05, 125.93, 122.6, 69.9, 69.5, 68.4, 53.4, 39.9, 25.76 (2C).

<u>HR-MS (ESI+):</u> m/z calculated for $[C_{21}H_{20}ClN_6O_{10}Re + Na]^+$: 759.03574, found: 759.03574, error: 0.8 ppm.

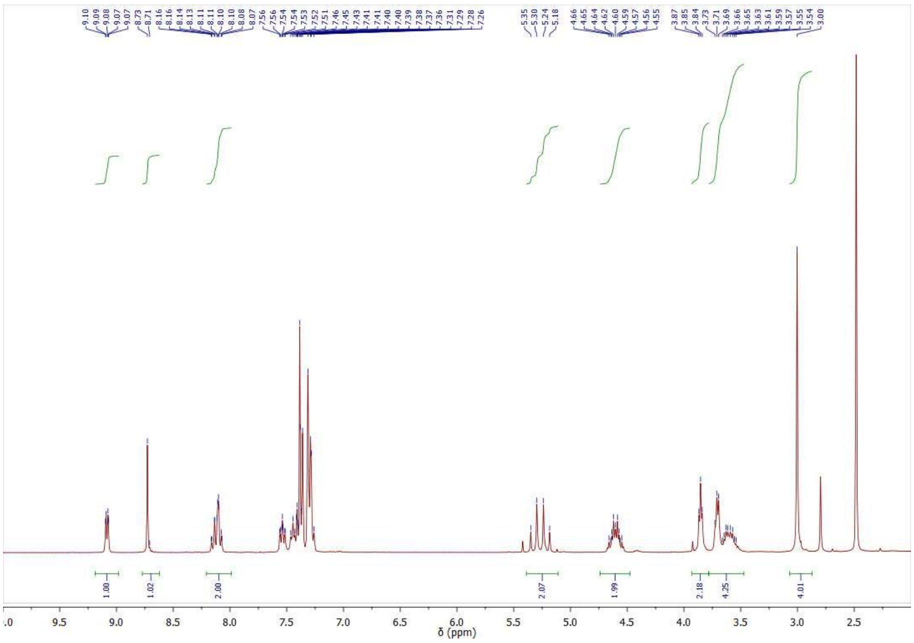

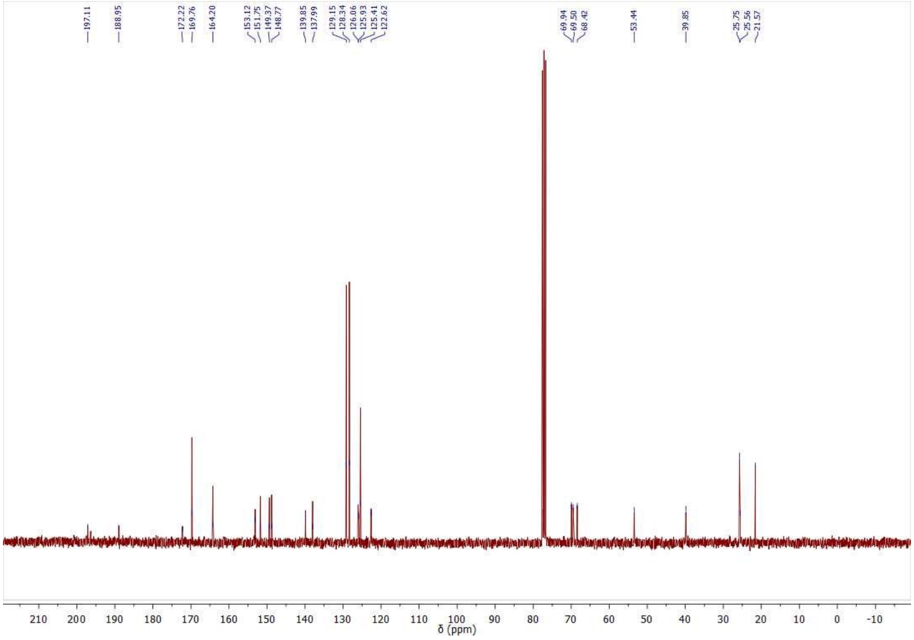

2.3.2 Synthesis of the ligand-imidazole moieties

*General procedure for compounds **11a-d***

The carboxylic acid (0.5-0.6 mmol, 1 equiv.), EDC·HCl (1.5 equiv.) and HOBt·H2O (1.5 equiv.) were dissolved in dry DMF (2.6 mL) under argon. After 5-10 minutes stirring, the amine (1 equiv.) and DIEA (260 µL, 1.5 mmol, 3 equiv.) were added and reaction mixture was stirred overnight at room temperature. Solvent was removed by rotary evaporation and the resulting sticky solid was taken up in a 1:1 mixture of EtOAc and saturated aqueous solution of $NaHCO_3$. After decantation, the organic layer was washed with brine, dried over anhydrous Na_2SO_4, filtered and concentrated. The crude was purified by automated silica gel flash column chromatography (5% MeOH in DCM) to give the desired compound as a white solid in 40-74% yields.

*C0-Trityl (**11a**)*

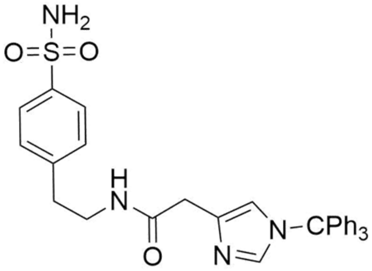

According to the general procedure, with 1-(triphenylmethyl)-1H-imidazole-4-acetic acid (221.7 mg, 0.60 mmol, 1.2 equiv.), and 4-(2-aminoethyl)benzene sulfonamide (100.1 mg, 0.50 mmol, 1 equiv.).

Yield: 40% (109.1 mg, 0.20 mmol).

^{1}H-NMR (400 MHz, CD_3OD): δ (ppm) 7.79 (d, J=8.5, 2H), 7.41-7.33 (m, 12H), 7.16 (ddt, J = 5.1, 2.3, 1.2, 6H), 6.85 (s, 1H), 3.43 (t, J = 7.2, 2H), 3.41 (d, J = 1.7, 2H), 2.84 (t, J = 7.3, 2H).

HR-MS (ESI+): m/z calculated for $[C_{32}H_{30}N_4O_3S+H]^+$: 551.21114, found: 551.21106, error: -0.1 ppm.

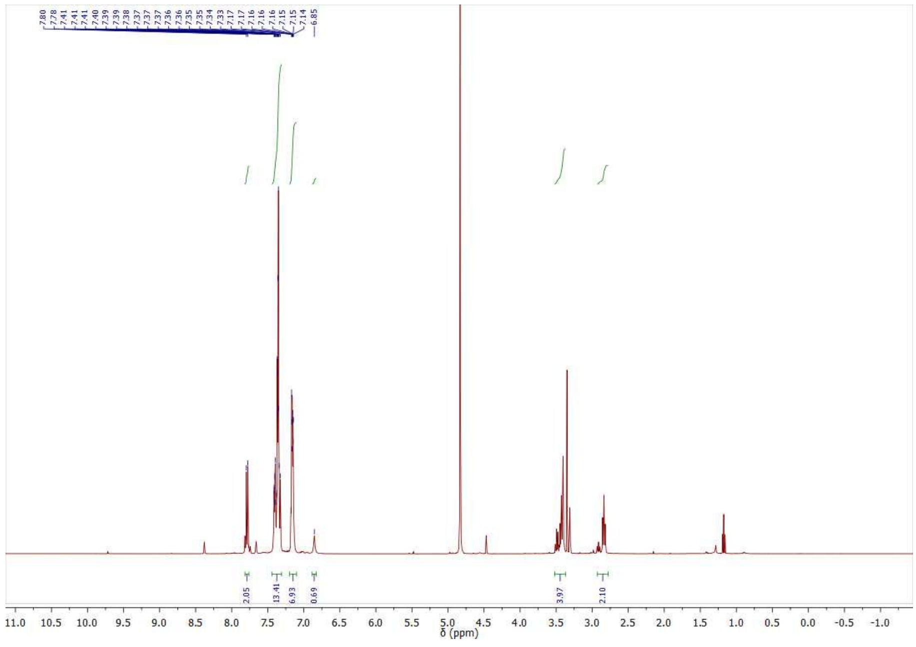

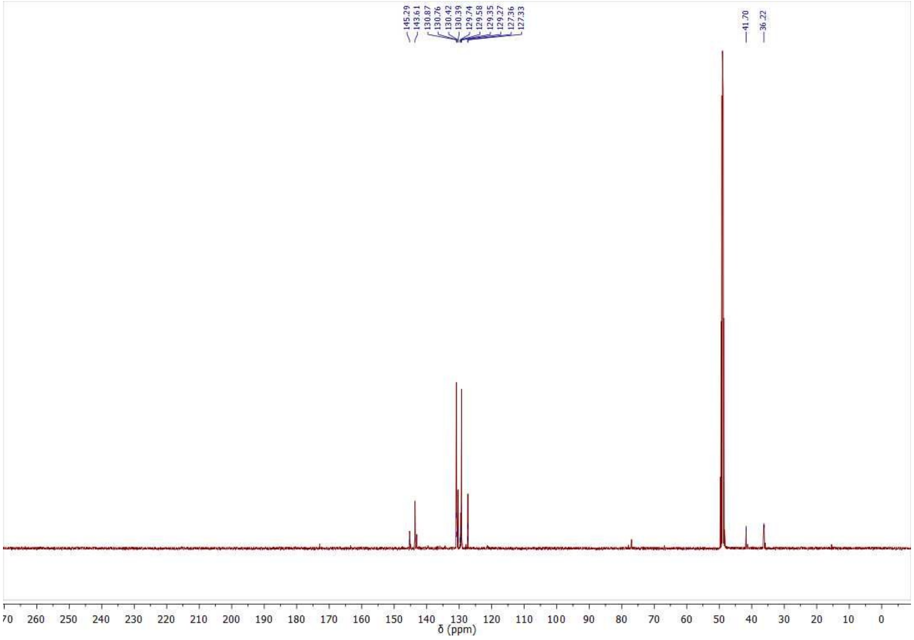

*C3-Boc (**11b**)*

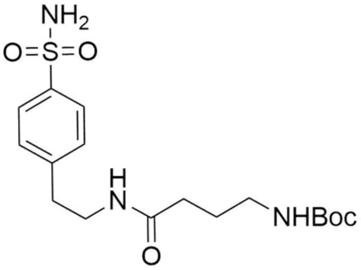

According to the general procedure, with Boc-4-aminobutanoic acid (104.6 mg, 0.51 mmol, 1 equiv.), and 4-(2-aminoethyl)benzene sulfonamide (100.1 mg, 0.50 mmol, 1 equiv).

Yield: 68% (133.2 mg, 0.35 mmol).

^{1}H-NMR (300 MHz, CD_3OD): δ (ppm) 7.83 (d, J = 8.2, 2H), 7.40 (d, J = 8.2, 2H), 3.43 (t, J = 7.1, 2H), 3.00 (t, J = 6.8, 2H), 2.88 (t, J = 7.1, 2H), 2.16 (t, J = 7.5, 2H), 1.70 (quintet, J = 7.2, 2H), 1.43 (s, 9H).

^{13}C-NMR (75 MHz, CD_3OD): δ (ppm) 175.5, 158.4, 145.3, 142.9, 130.4 (2C), 127.2 (2C), 79.9, 41.3, 40.7, 36.2, 34.3, 28.8 (3C), 27.2.

HR-MS (ESI+): m/z calculated for $[C_{17}H_{27}N_3O_5S+Na]^+$: 408.15636, found: 408.15718, error: 2.0 ppm.

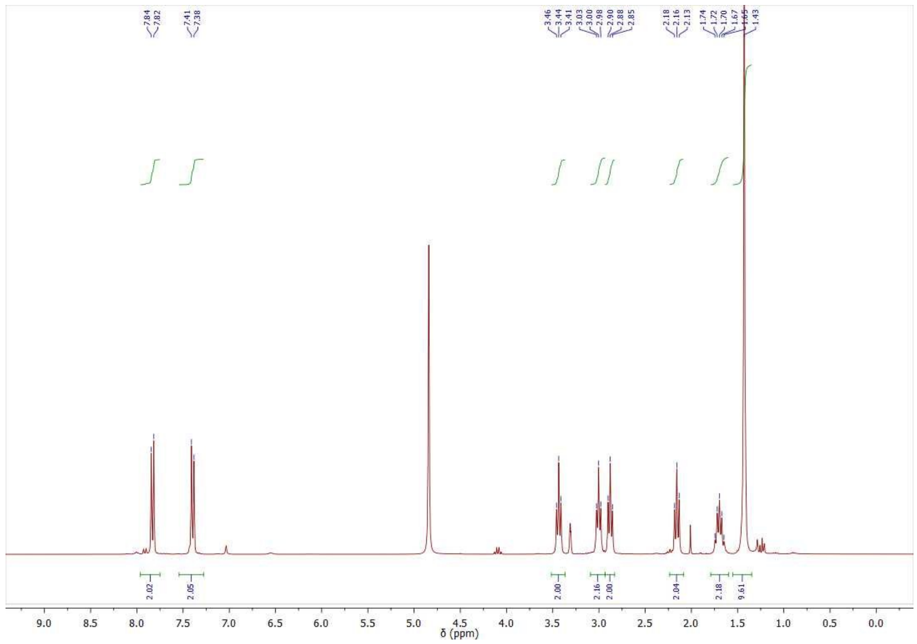

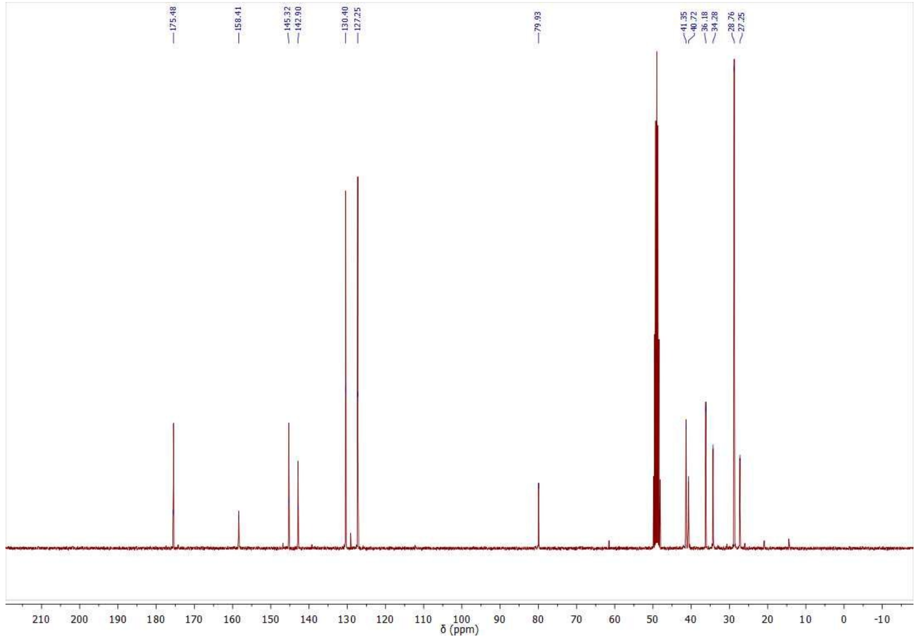

*C6-Boc (**11c**)*

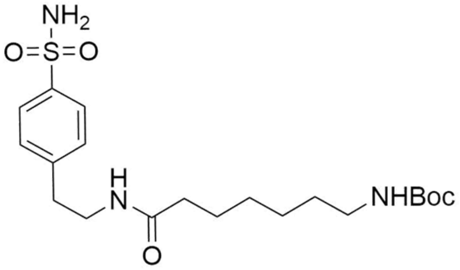

According to the general procedure, with Boc-7-aminoheptanoic acid (101.8 mg, 0.51 mmol, 1 equiv.), and 4-(2-aminoethyl)benzene sulfonamide (249.7 mg, 1.25 mmol, 2.5 equiv.).

<u>Yield:</u> 67% (146.1 mg, 0.34 mmol).

^{1}H-NMR (400 MHz, CD_3OD): δ (ppm) 7.84 (d, J = 8.5, 2H), 7.41 (d, J = 8.6, 2H), 3.46 (t, J = 7.2, 2H), 3.02 (t, J = 7.0, 2H), 2.90 (t, J = 7.1, 2H), 2.15 (t, J = 7.5, 2H), 1.57 (t, J = 7.1, 2H), 1.47-1.44 (m, 11H), 1.30 (dt, J = 6.7, 3.1, 4H).

^{13}C-NMR (101 MHz, CD_3OD): δ (ppm) 176.2, 158.5, 145.4, 143.1, 130.4 (2C), 127.3 (2C), 79.8, 41.3 (2C), 37.0, 36.3, 30.8, 29.9, 28.8 (3C), 27.5, 26.9.

HR-MS (ESI+): m/z calculated for $[C_{20}H_{33}N_3O_5S+Na]^+$: 450.20331, found: 450.20351, error: 0.4 ppm.

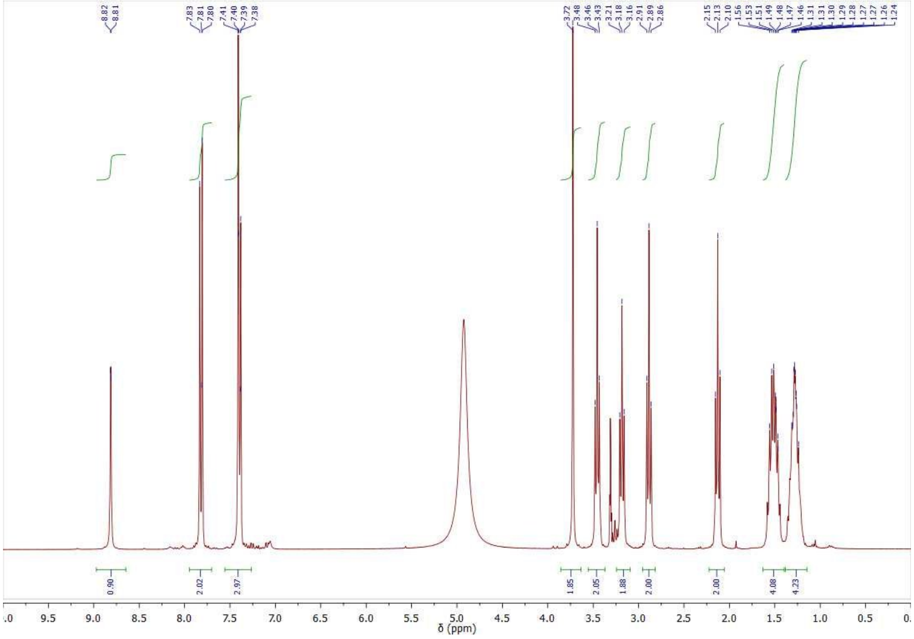

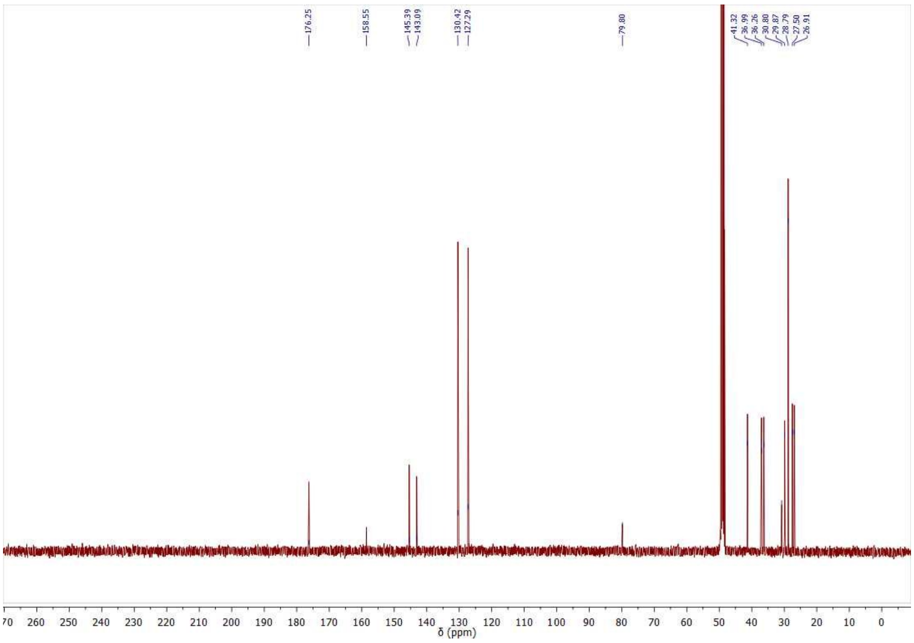

*C5-Boc (**11d**)*

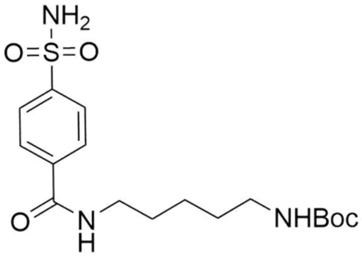

According to the general procedure, with 4-carboxybenzenesulfonamide (120.8 mg, 0.6 mmol, 1.25 equiv.), and *N*-Boc-1,5-diaminopentane (0.1 mL, 0.48 mmol, 1 equiv.).

<u>Yield:</u> 74% (173.8 mg, 0.45 mmol).

<u>^{1}H-NMR (400 MHz, CD_3OD):</u> δ (ppm) 7.98-7.93 (m, 4H), 3.39 (t, J = 7.1, 2H), 3.05 (t, J = 6.9, 2H), 1.65 (quintet, J = 7.3, 2H), 1.56-1.49 (m, 2H), 1.42-1.38 (m, 11H).

<u>^{13}C-NMR (101 MHz, CD_3OD)</u>: δ (ppm) 168.71, 158.53, 147.53, 139.16, 128.90 (2C), 127.26 (2C), 79.80, 41.02 (2C), 30.03 (2C), 28.77 (3C), 25.18.

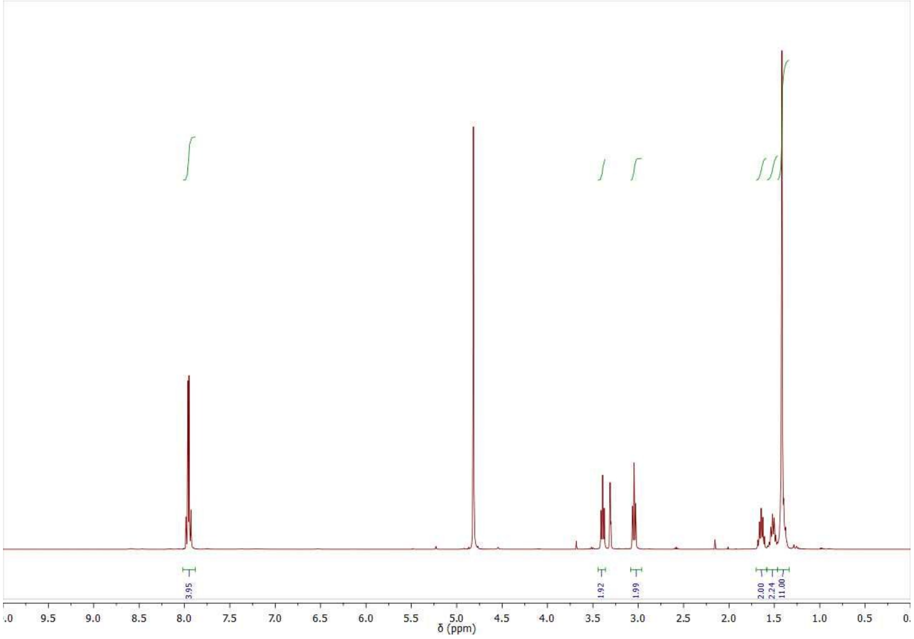

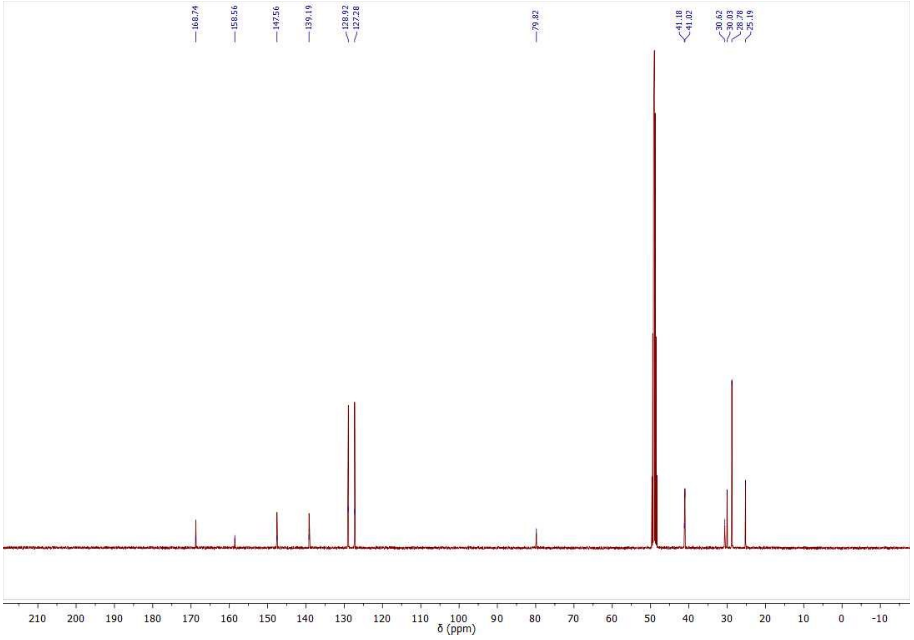

*General procedure for compounds **12b-d***

The Boc-protected compound **11** (0.25 mmol, 1 equiv.) was dissolved in DCM (1 mL), and TFA (1 mL) was added drop wise. The solution was stirred for 1 h at room temperature, then precipitated in cold Et2O (40 mL) to give the deprotected amine as a white solid. 1-(triphenylmethyl)-1H-imidazole-4-acetic acid (1.2 equiv.), EDC·HCl (1.5 equiv.) and HOBt ·H2O (1.5 equiv.) were dissolved in dry DMF (1.3 mL) under argon. After 5-10 minutes stirring, the solution was added to the deprotected amine. DIEA (3 equiv.) was added and reaction mixture was stirred overnight at room temperature. Solvent was removed by rotary evaporation and the resulting sticky solid was taken up in a 1:1 mixture of EtOAc and saturated aqueous solution of NaHCO3. After decantation, the organic layer was washed with brine, dried over anhydrous Na2SO4, filtered and concentrated. The crude was purified by automated silica gel flash column chromatography (5% MeOH in DCM). The resulting oil was co-evaporated with Et2O until obtention of the desired compound as a white solid. Yields 34-54%.

(88.7 mg, 0.14 mmol, 54%).

*C3-Trityl (**12b**)*

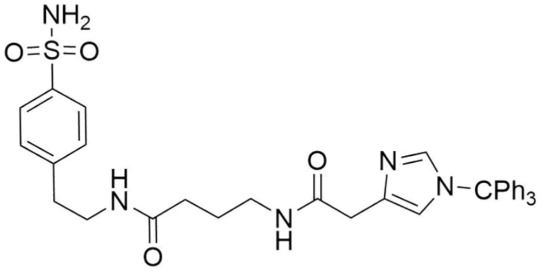

According to the general procedure, with Boc-protected compound **11b** (100 mg, 0.26 mmol, 1 equiv.)

Yield: 54% (88.7 mg, 0.14 mmol).

^{1}H-NMR (400 MHz, CD_3OD): δ (ppm) 7.81 (d, J = 8.4, 2H), 7.43 (s, 1H), 7.36 (ddt, J = 5.9, 2.3, 2.8, 10H), 7.17-7.14 (m, 5H), 6.87 (s, 1H), 3.43 (t, J = 7.1, 4H), 3.11 (dd, J = 8.8, 5.0, 2H), 2.86 (t, J = 7.2, 2H), 2.13 (t, J = 7.4, 2H), 1.70 (t, J = 7.2, 2H).

^{13}C-NMR (75 MHz, CD_3OD): δ (ppm) 175.47, 158.40, 145.31, 142.89, 130.40, 127.24, 79.92, 41.35, 40.72, 36.18, 34.28, 28.76, 27.25.

HR-MS (ESI+): m/z calculated for $[C_{36}H_{37}N_5O_4S+H]^+$: 636.26390, found: 636.26423, error: 0.5 ppm.

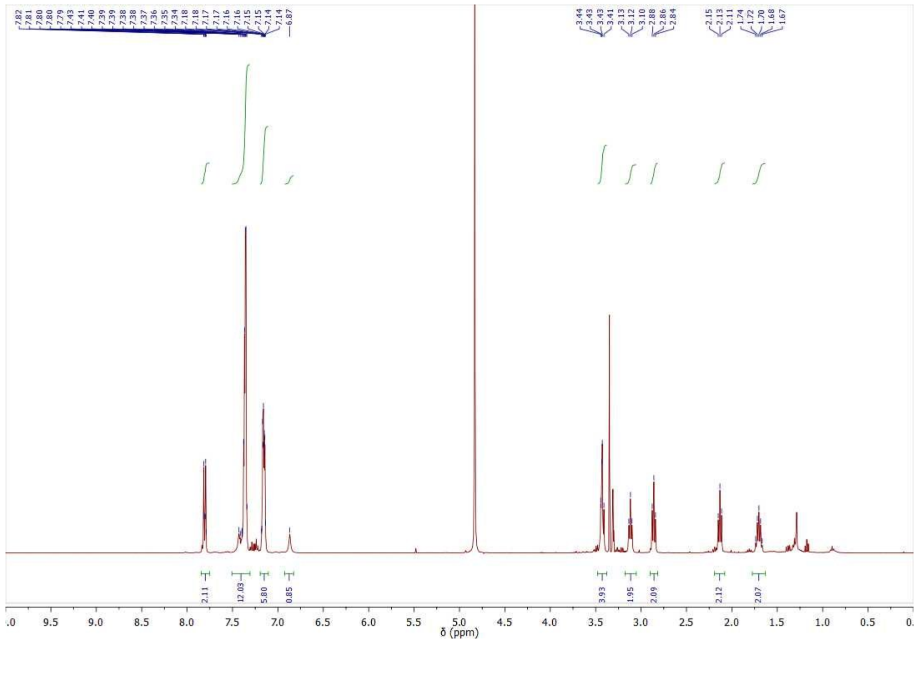

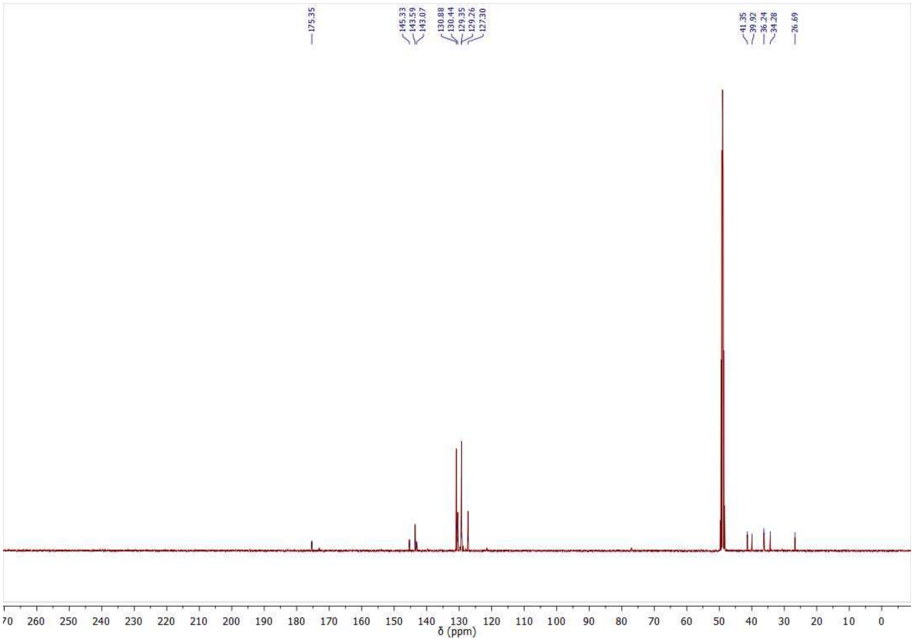

*C6-Trityl (**12c**)*

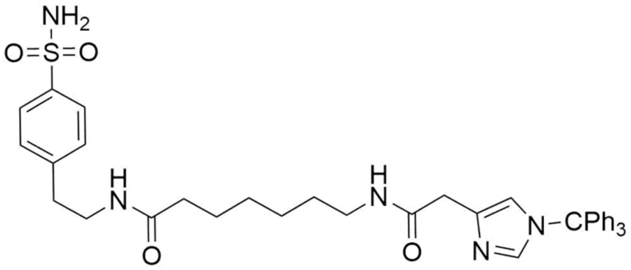

According to the general procedure, with Boc-protected compound **11c** (120 mg, 0.28 mmol, 1 equiv.).

Yield: 42% (79.8 mg, 0.12 mmol).

^{1}H-NMR (400 MHz, CD_3OD): δ (ppm) 7.84-7.81 (m, 2H), 7.40-7.34 (m, 12H), 7.16-7.14 (m, 6H), 6.85 (s, 1H), 3.43 (d, J = 7.1, 4H), 3.15 (t, J = 6.9, 2H), 2.87 (t, J = 7.1, 3H), 2.11 (t, J = 7.4, 3H), 1.51 (ddd, J = 30.5, 15.9, 7.1, 5H), 1.30-1.21 (m, 6H).

^{13}C-NMR (101 MHz, CD_3OD): δ (ppm) 176.14, 145.35, 143.61, 143.07, 130.86, 130.41, 129.33, 129.24, 127.28, 41.28, 40.64, 40.45, 36.95, 36.23, 30.15, 29.75, 27.51, 26.84.

HR-MS (ESI+): m/z calculated for $[C_{39}H_{43}N_5O_4S+H]^+$: 678.31085, found: 678.31146, error: 0.9 ppm.

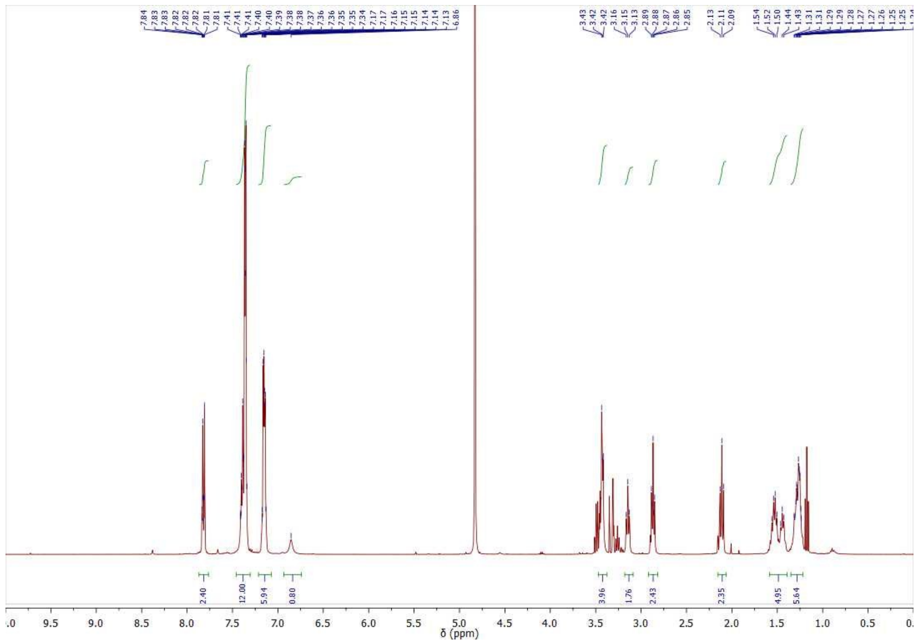

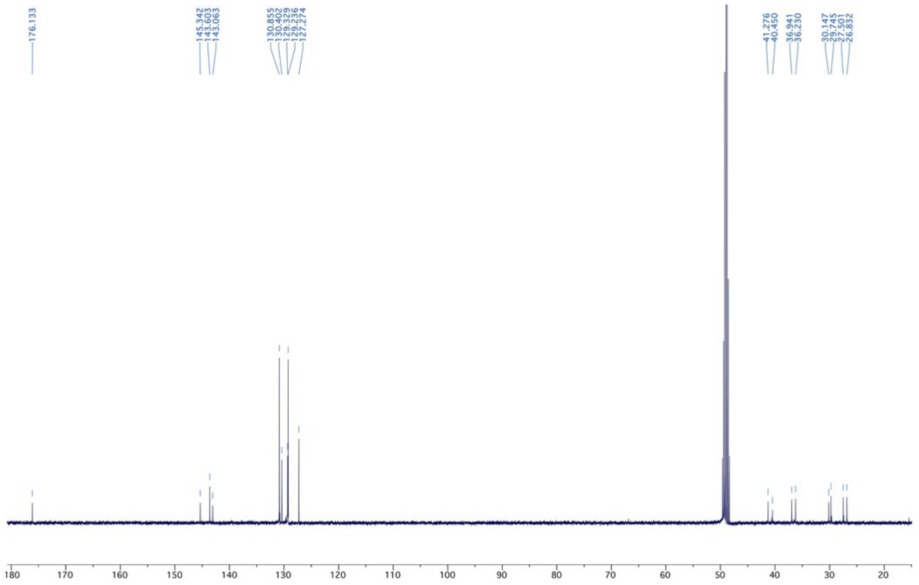

*C5-Trityl (**12d**)*

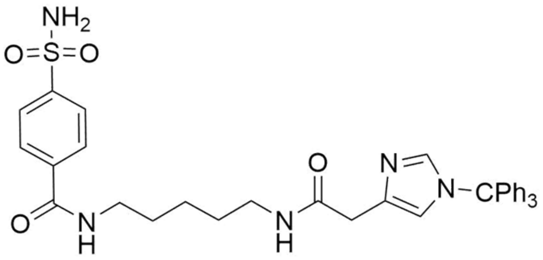

According to the general procedure, with Boc-protected compound **11d** (112.7 mg, 0.29 mmol, 1 equiv.).

<u>Yield:</u> 34% (63.4 mg, 0.10 mmol).

<u>^{1}H-NMR (400 MHz, CD_3OD):</u> δ (ppm) 7.95 (q, J = 7.1, 4H), 7.41 (s, 1H), 7.37 (dt, J = 4.3, 2.8, 9H), 7.15 (dd, J = 6.8, 3.0, 6H), 6.85 (s, 1H), 3.43 (s, 2H), 3.36 (t, J = 7.1, 2H), 3.19 (t, J = 7.0, 2H), 1.67-1.59 (m, 2H), 1.53 (dt, J = 14.4, 7.2, 2H), 1.38 (dt, J = 15.4, 7.7, 2H).

^{13}C-NMR (101 MHz, CD_3OD): δ (ppm) 172.89, 168.72, 147.61, 143.61, 139.18, 130.87, 129.35, 129.25, 128.93, 127.30, 121.28, 77.00, 41.00, 40.38, 29.99, 29.94, 25.24.

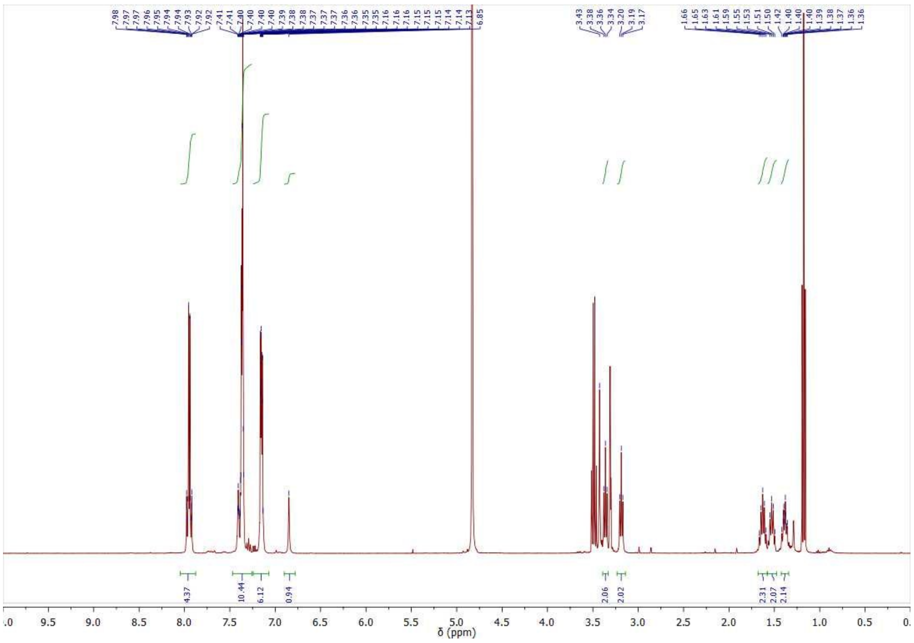

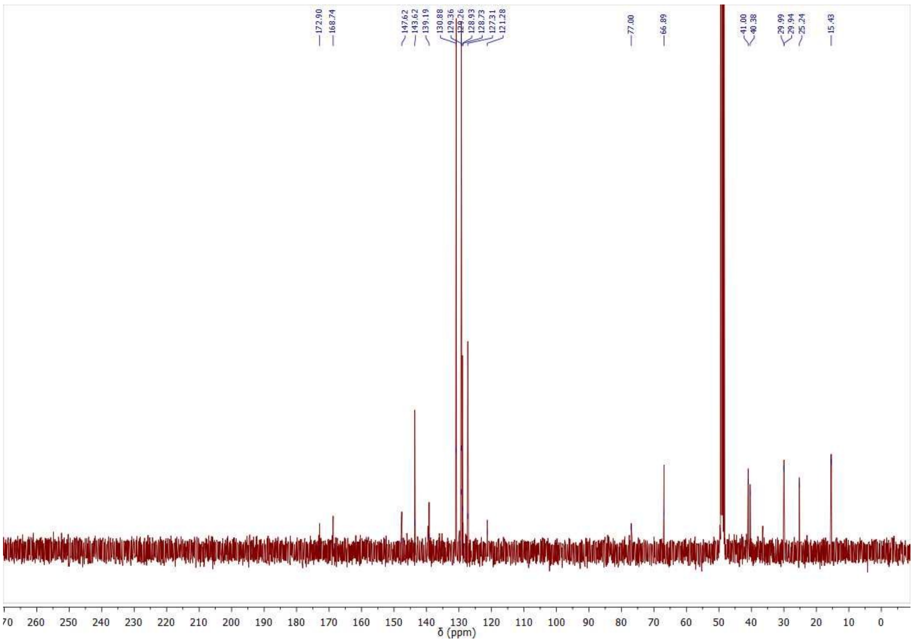

*General procedure for compounds **13a-d***

The trityl imidazole derivative **12** (0.091-0.095 mmol, 1 equiv.) was dissolved in DCM (0.8 mL). TFA (0.2 mL) was added drop wise, followed by TIS (1 equiv.). The mixture was stirred for 1 hour at room temperature, then precipitated in Et_2O to give the desired compound as a white solid/oil containing traces of water, in 85-93% yields.

*C0-imidazole (**13a**)*

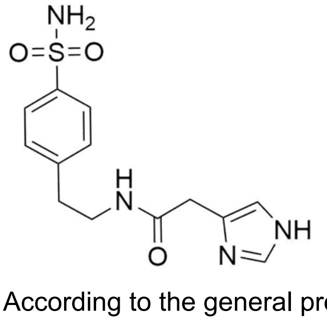

According to the general procedure for the deprotection of the imidazole with compound **11a**.

Yield: 93% (34.6 mg of hydrated compound).

^{1}H-NMR (300 MHz, CD_3OD): δ (ppm) 8.80 (s, 1H), 7.80 (d, J = 8.4, 2H), 7.39 (d, J = 8.4, 2H), 7.35 (s, 1H), 3.67 (s, 2H), 3.49 (t, J = 7.2, 2H), 2.90 (t, J = 7.2, 2H).

^{13}C-NMR (75 MHz, CD_3OD): δ (ppm) 169.9, 145.2, 143.1, 135.0, 130.4 (2C), 129.2, 127.3 (2C), 118.5, 41.6, 36.1, 32.0.

HR-MS (ESI+): m/z calculated for $[C_{13}H_{16}N_4O_3S+H]^+$: 309.10159, found: 309.10170, error: 0.4 ppm.

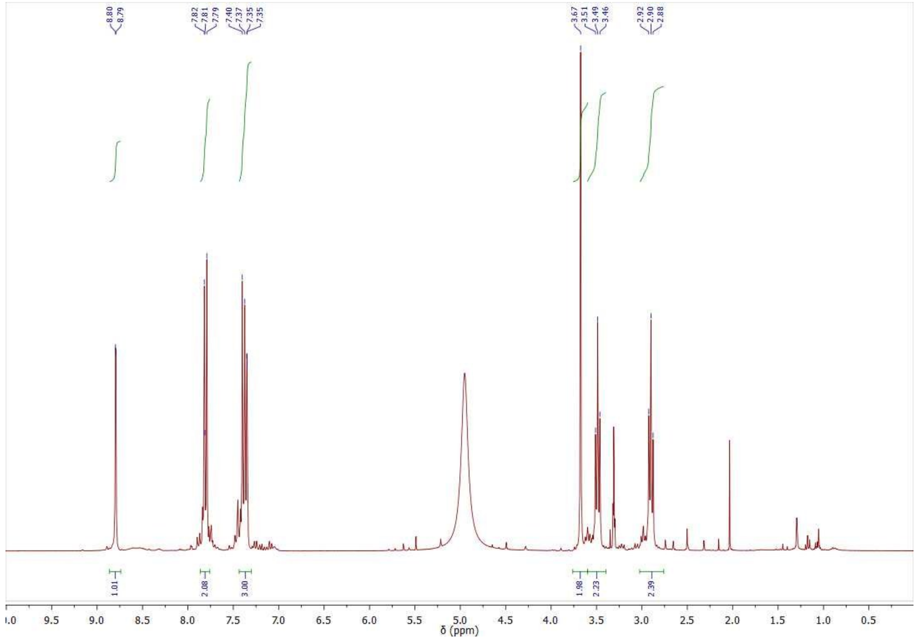

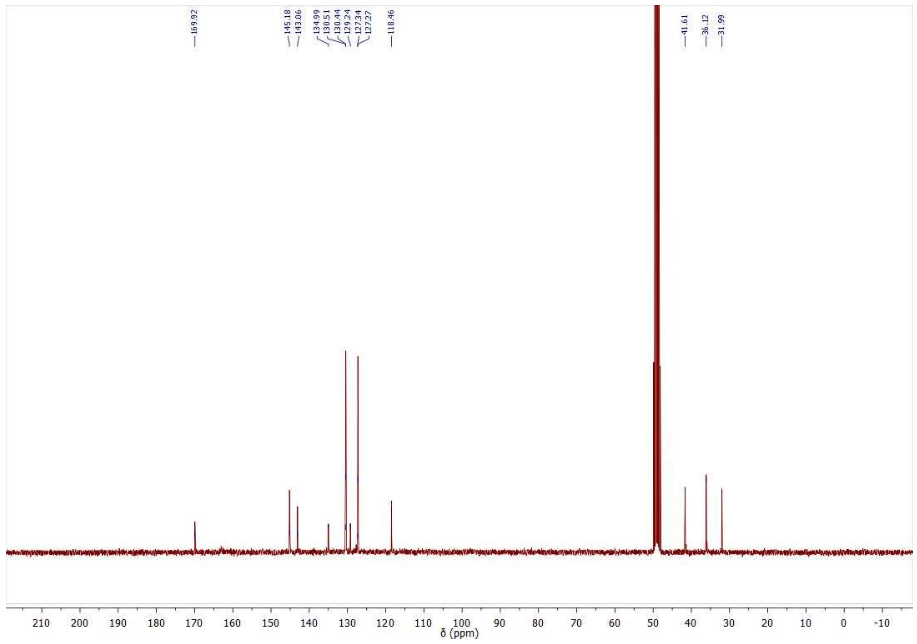

*C3-imidazole (**13b**)*

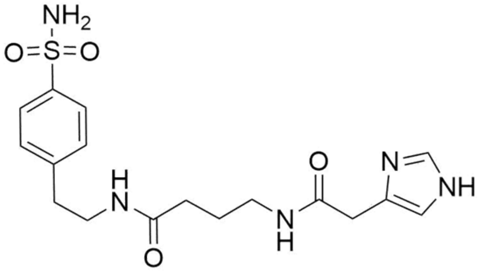

According to the general procedure for the deprotection of the imidazole with compound **12b**.

<u>Yield:</u> 85% (39.7 mg of hydrated compound).

<u>^{1}H-NMR (300 MHz, CD_3OD):</u> δ (ppm) 8.82 (d, J = 1.4, 1H), 7.81 (d, J = 8.5, 2H), 7.39 (d + s, J = 8.5, 2H + 1H), 3.72 (s, 2H), 3.45 (t, J = 7.1, 2H), 3.13 (t, J = 7.1, 2H), 2.88 (t, J = 7.1, 2H), 2.17 (t, J = 7.4, 2H), 1.73 (quintet, J = 7.2, 2H).

<u>^{13}C-NMR (75 MHz, CD_3OD):</u> δ (ppm) 175.3, 169.9, 145.4, 142.9, 135.0, 130.49 (2C), 129.2, 127.2 (2C), 118.6, 41.3, 40.1, 36.2, 34.3, 32.0, 26.6.

<u>HR-MS (ESI+)</u>: m/z calculated for $[C_{17}H_{23}N_5O_4S+H]^+$: 394.15435, found: 394.15448, error: 0.3 ppm.

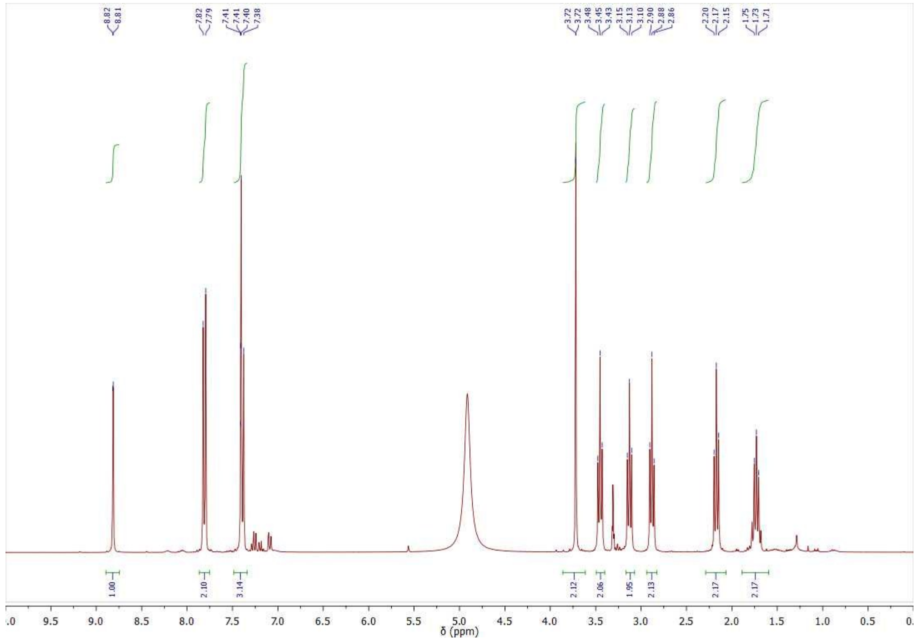

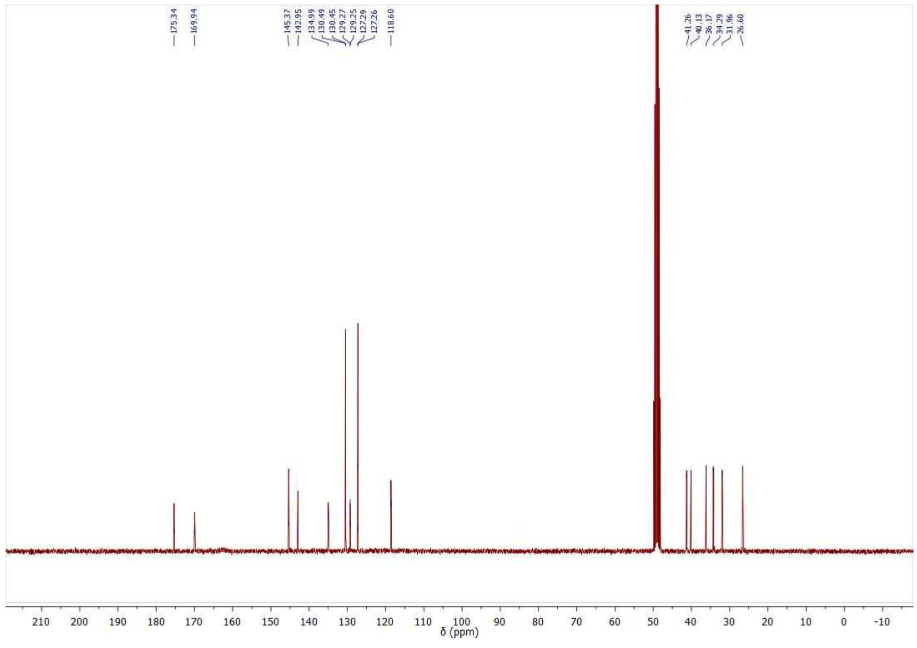

*C6-imidazole (**13c**)*

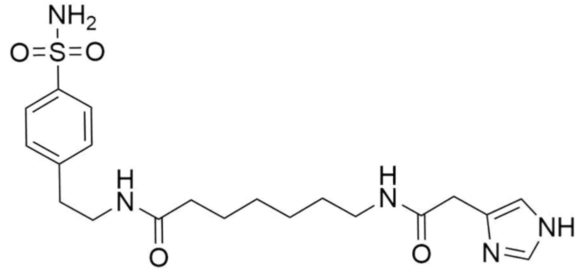

.

According to the general procedure for the deprotection of the imidazole with compound **12c**.

Yield: 88% (35.6 mg of hydrated compound, 0.070 mmol).

^{1}H-NMR (300 MHz, CD_3OD): δ (ppm) 8.81 (s, 1H), 7.82 (d, J = 8.4, 2H), 7.41-7.38 (d + s, J = 8.3, 2H + 1H), 3.72 (s, 2H), 3.46 (t, J = 7.0, 2H), 3.18 (t, J = 7.1, 2H), 2.89 (t, J = 7.0, 2H), 2.13 (t, J = 7.4, 2H), 1.51 (tt, J = 13.7, 6.9, 2H + 2H), 1.33-1.24 (m, 4H, 2H + 2H).

^{13}C-NMR (75 MHz, CD_3OD): δ (ppm) 176.2, 169.8, 145.4, 143.0, 135.0, 130.46 (2C), 129.4, 127.2 (2C), 118.5, 41.2, 40.7, 36.9, 36.1, 32.0, 30.1, 29.7, 27.6, 26.8.

HR-MS (ESI+): m/z calculated for $[C_{20}H_{29}N_5O_4S+H]^+$: 436.20130, found: 436.20148, error: 0.4 ppm.

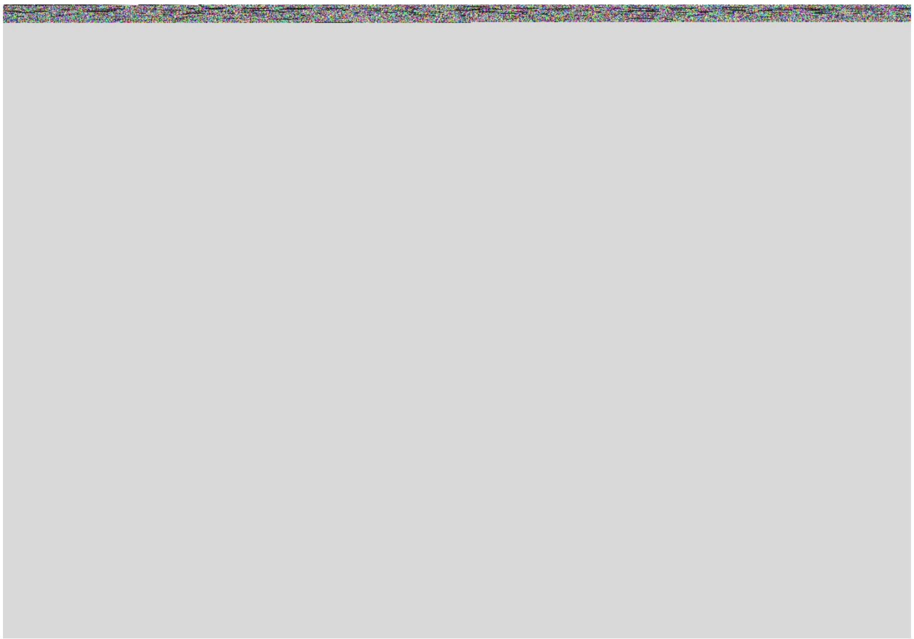

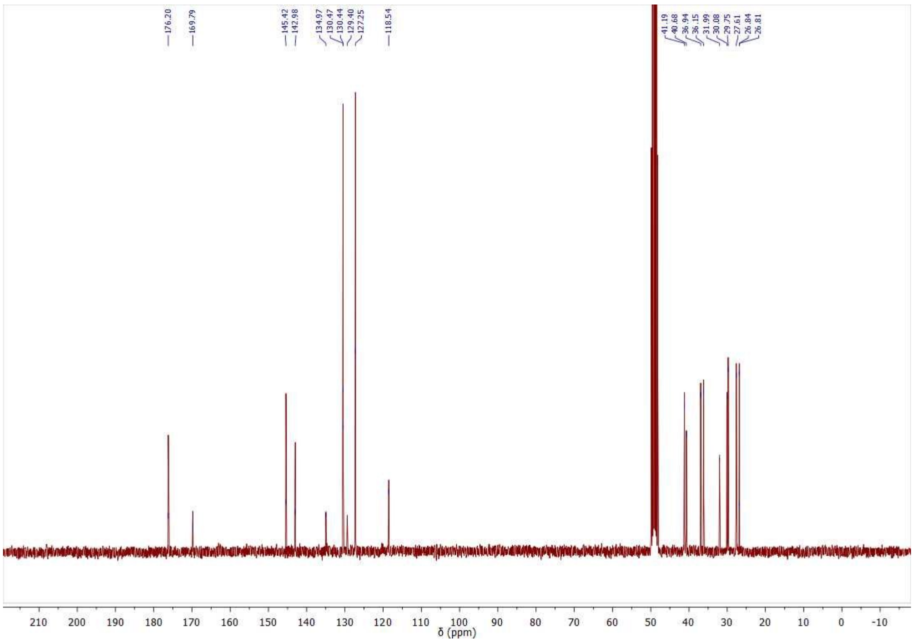

*C5-imidazole (**13d**)*

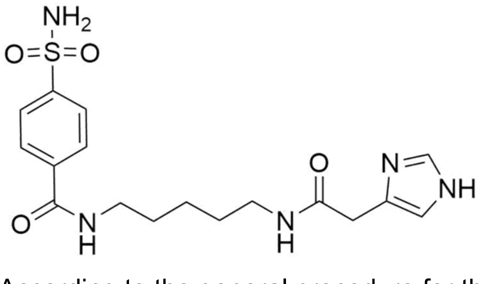

According to the general procedure for the deprotection of the imidazole with compound **12d**.

Yield: 90% (43.2 mg of hydrated compound, 0.095 mmol).

^{1}H-NMR (300 MHz, CD_3OD): δ (ppm) 8.81 (s, 1H), 7.95 (s, 4H), 7.39 (s, 1H), 3.71 (s, 2H), 3.39 (t, J = 6.7, 2H), 3.22 (t, J = 6.3, 2H), 1.61 (td, J = 13.6, 6.7, 2H + 2H), 1.41 (t, J = 6.6, 2H).

^{13}C-NMR (75 MHz, CD_3OD): δ (ppm) 169.80, 168.74, 147.54, 139.12, 134.96, 129.36, 128.92 (2C), 127.27 (2C), 118.49, 40.94, 40.58, 31.96, 29.99-29.91 (2C), 25.27.

HR-MS (ESI+): m/z calculated for $[C_{17}H_{23}N_5O_4S+H]^+$: 394.15435, found: 394.15443, error: 0.2 ppm.

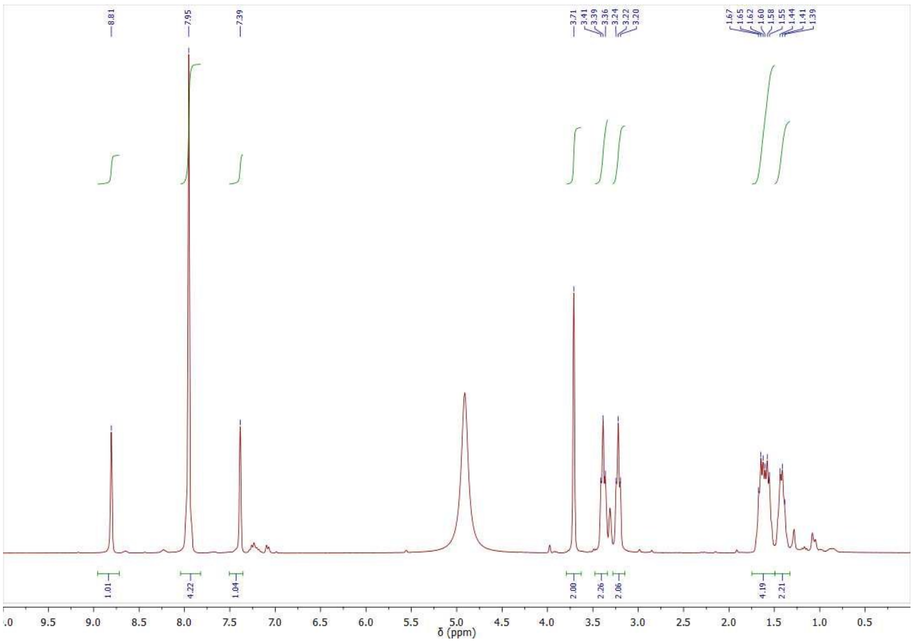

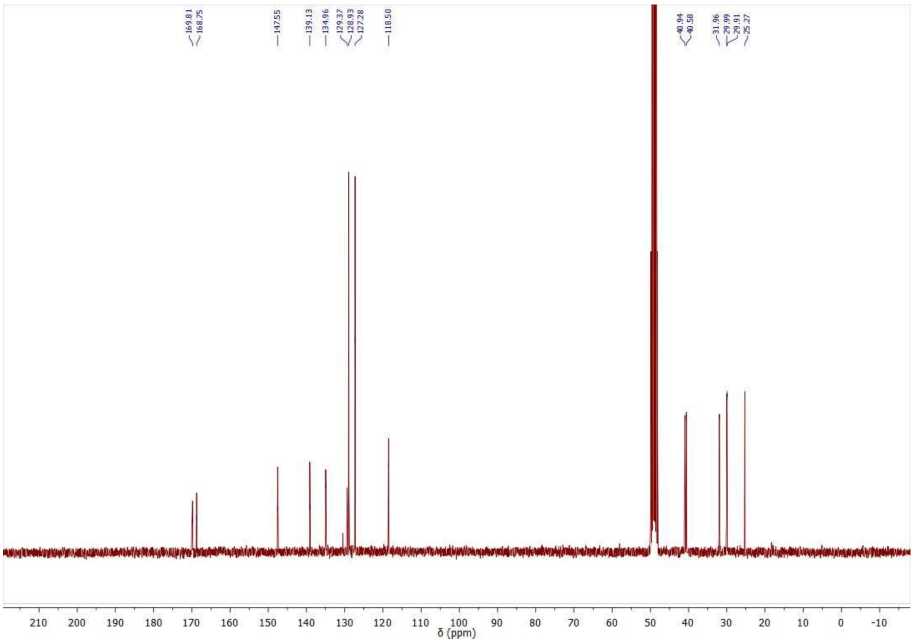

2.3.3 Synthesis of the SCoMPI-LDAI

*General procedure for the synthess of LDAI-SCoMPI (**14a-d**)*

The imidazole **13** (1.1 equiv.) and the rhenium complex **10** (25 µmol, 1 equiv.) were dissolved in dry DMF (2 mL) under argon. Dry pyridine (1.1 equiv.) was added and the reaction mixture was stirred overnight at RT. Solvent was removed by rotary evaporation and the resulting crude was purified by HPLC (XBridgeTM Prep C18 OBDTM column, 25 to 100 % TFA-free ACN in 10 mM aqueous NH_4OAc pH 7.0 in 30 min, 14 mL/min). NH_4OAc was removed by repeated lyophilization cycles.

*C0-SCoMPI (**14a**)*

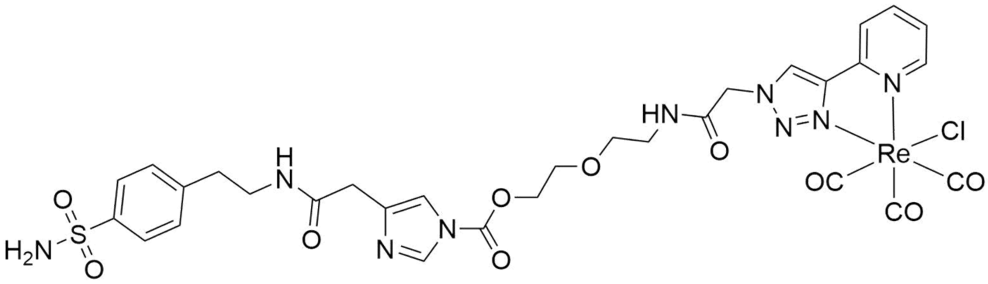

According to the general procedure, with C0-imidazole **13a** (8.6 mg, 27.9 µmol, 1.1 equiv.) and the rhenium complex **10** (18.6 mg, 25.2 µmol, 1 equiv.)

HPLC: t_R = 11.4 min, purity: 97%.

^{1}H-NMR (400 MHz, $CDCl_3$: CD_3OD): δ (ppm) 8.96 (d, J = 5.5, 1H), 8.82 (s, 1H), 8.14 (s, 1H), 8.11-8.05 (m, 2H), 7.74 (d, J = 8.3, 2H), 7.50 (ddd, J = 7.1, 5.4, 1.8, 1H), 7.36 (s, 1H), 7.27 (d, J = 8.3, 2H), 5.25 (q, J = 22.2, 2H), 4.55 (m, 2H, under water signal), 3.84-3.82 (m, 2H), 3.64 (t, J = 5.3, 2H), 3.48-3.41 (m + d + s, 2H + 2H + 2H), 2.84 (t, J = 7.0, 2H).

HR-MS (ESI+): m/z calculated for $[C_{30}H_{31}ClN_9O_{10}ReS+Na]^+$: 952.10249, found: 952.10229, error: -0.2 ppm.

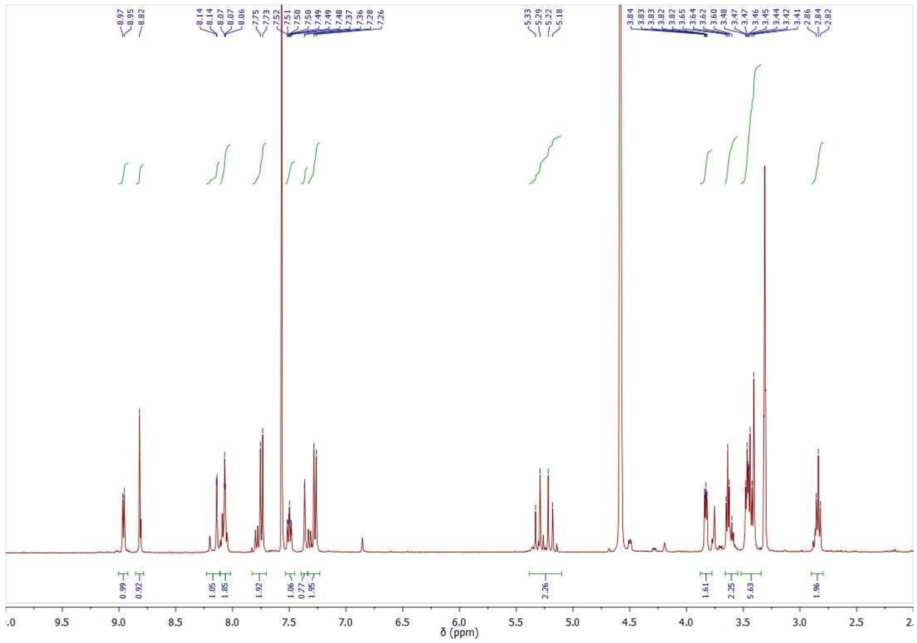

*C3-SCoMPI (**14b**)*

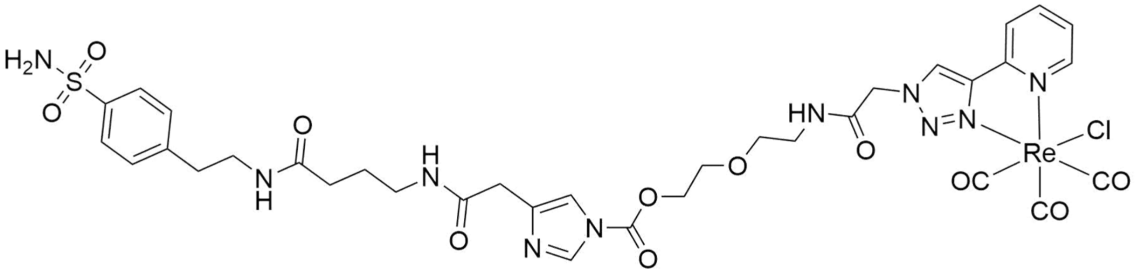

According to the general procedure, with C3-imidazole **13b** (11.9 mg, 30.2 µmol, 1.1 equiv.) and the rhenium complex **10** (19.1 mg, 25.9 µmol, 1 equiv.).

HPLC: t_R = 10.3 min, purity: 94%.

^{1}H-NMR (400 MHz, $CDCl_3$: CD_3OD): δ (ppm) 8.96 (dt, J = 5.5, 1.1, 1H), 8.83 (s, 1H), 8.17 (s, 1H), 8.13-8.06 (m, 2H), 7.78 (d, J = 8.4, 2H), 7.51 (ddd, J = 6.9, 5.6, 2.1, 1H), 7.45 (s, 1H), 7.33 (d, J = 8.5, 2H), 5.26 (q, J = 22.4, 2H), 4.58-4.56 (m, 2H, partly hidden by water signal), 3.83-3.80 (m, 2H), 3.63 (t, J = 5.2, 2H), 3.47-3.40 (m + d + s, 2H + 2H + 2H), 3.07 (t, J = 7.0, 2H), 2.85 (t, J = 7.0, 2H), 2.10 (t, J = 7.3, 2H), 1.73-1.64 (m, 2H).

HR-MS (ESI+): m/z calculated for $[C_{34}H_{38}ClN_{10}O_{11}ReS+Na]^+$: 1037.15525, found: 1037.15545, error: 0.2 ppm.

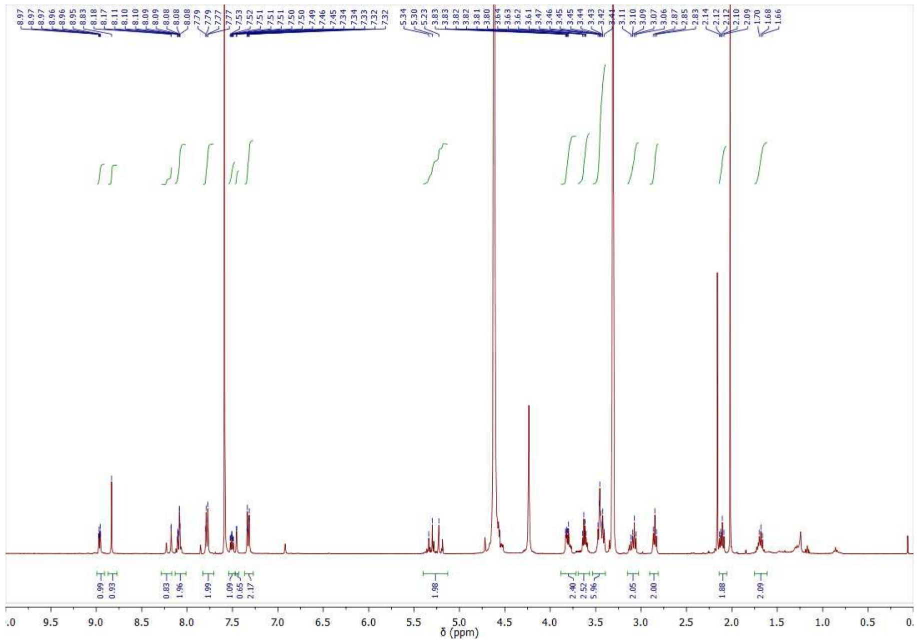

*C6-SCoMPI (**14c**)*

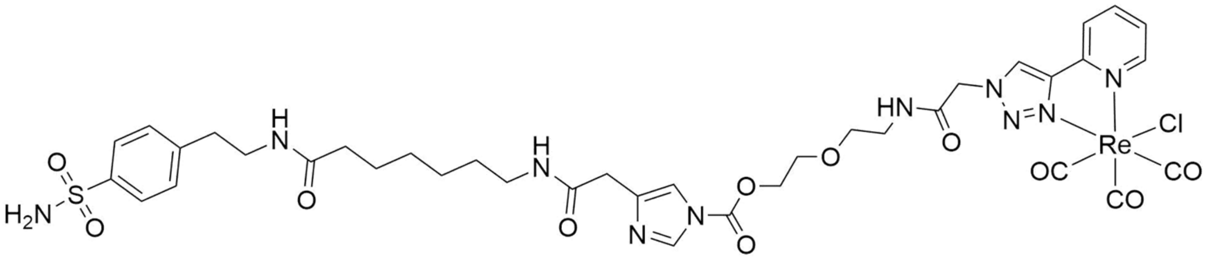

According to the general procedure, with C6-imidazole **13c** (12.8 mg, 29.4 µmol, 1.1 equiv.) and the rhenium complex **10** (19.4 mg, 26.3 µmol, 1 equiv.).

HPLC: t_R = 11.6 min, purity: 94%.

^{1}H-NMR (400 MHz, CD_3OD : $CDCl_3$): δ (ppm) 8.96 (dd, J = 5.6, 0.5, 1H), 8.82 (s, 1H), 8.16 (s, 1H), 8.10-8.06 (m, 2H), 7.79 (dd, J = 8.5, 1.4, 2H), 7.50 (ddd, J = 7.0, 5.5, 2.0, 1H), 7.45 (s, 1H), 7.33 (d, J = 8.5, 2H), 5.25 (q, J=22.5, 2H), 4.56-4.55 (m, 2H, partly hidden by water signal), 3.82-3.80 (m, 2H,), 3.62 (t, J = 5.3, 2H), 3.44 (dt, J = 12.3, 6.2, m + d + s, 2H + 2H + 2H), 3.13 (t, J = 7.1, 2H), 2.85 (t, J = 7.1, 2H), 2.09 (t, J = 7.4, 2H), 1.51 (dt, J=14.4, 7.6, 2H), 1.43 (dt, J=14.6, 7.4, 2H), 1.28-1.19 (m, 4H).

HR-MS (ESI+): m/z calculated for $[C_{36}H_{44}ClN_{10}O_{11}ReS+Na]^+$: 1079.20220, found: 1079.2016, error: -0.6 ppm.

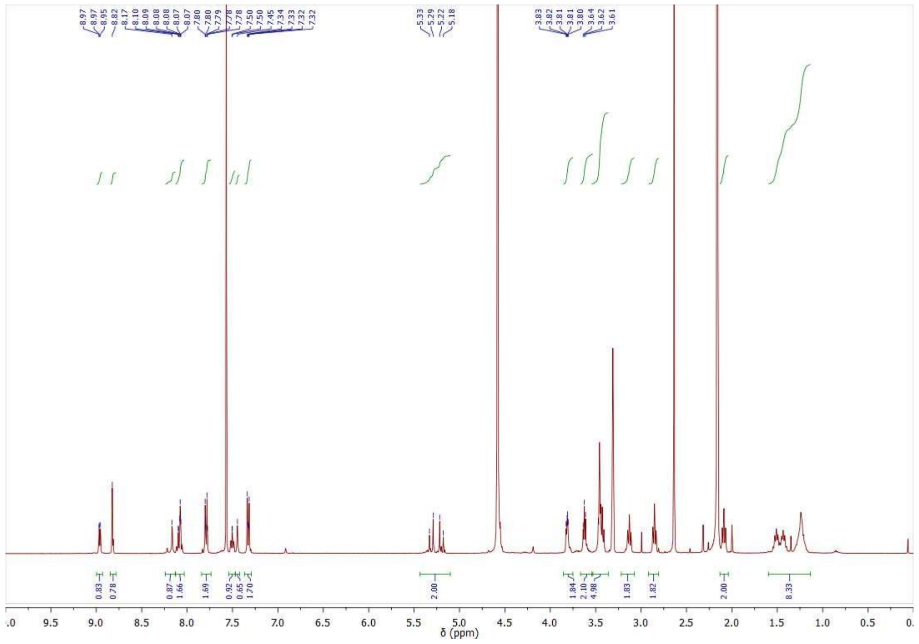

*C5-SCoMPI (**14d**)*

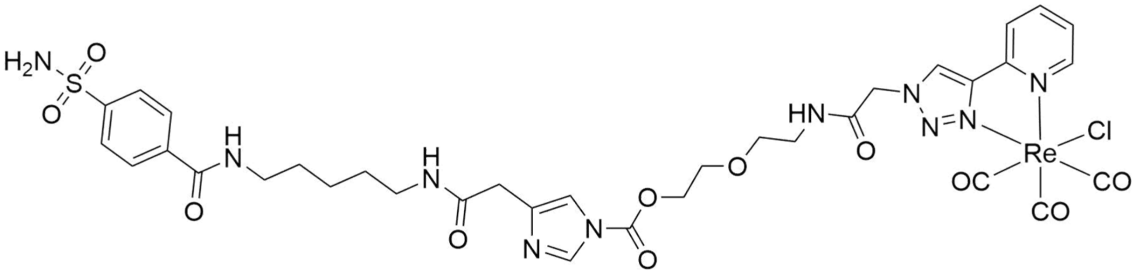

According to the general procedure, with C5-imidazole **13d** (12.4 mg, 31.5 µmol, 1.3 equiv.) and the rhenium complex **10** (18.3 mg, 24.8 µmol, 1 equiv.).

HPLC: t_R = 11.3 min, purity: 96%.

^{1}H-NMR (400 MHz, CD_3OD : $CDCl_3$): δ (ppm) 8.96-8.94 (m, 1H), 8.81 (s, 1H), 8.13 (s, 1H), 8.11-8.04 (m, 2H), 7.94-7.89 (m, 4H), 7.50 (ddd, J = 7.3, 5.5, 1.8, 1H), 7.42 (s, 1H), 5.25 (q, J = 23.0, 2H), 4.57-4.55 (m, 2H, partly hidden by water signal), 3.81 (td, J = 3.1, 2.3, 2H), 3.62 (t, J = 5.4, 2H), 3.45 (m + s, 2H + 2H), 3.38-3.33 (m, 2H), 3.18 (t, J = 7.0, 2H), 1.61 (dt, J = 14.6, 7.3, 2H), 1.52 (dd, J = 14.5, 6.9, 2H), 1.38 (dd, J = 16.1, 7.6, 2H).

HR-MS (ESI+): m/z calculated for $[C_{34}H_{38}ClN_{10}O_{11}ReS+Na]^+$: 1037.15525, found: 1037.15464, error: -0.6 ppm.

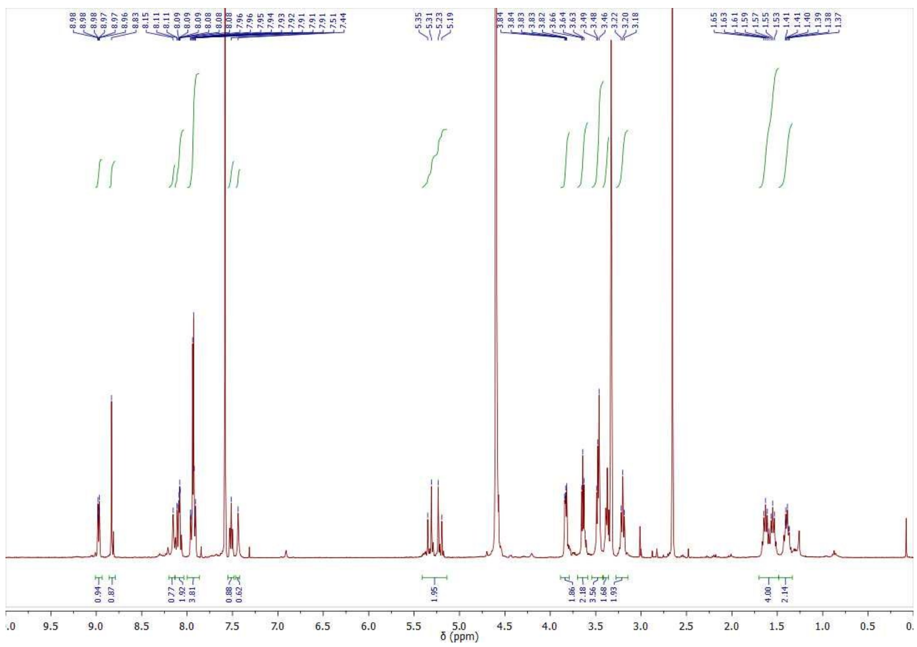

8.98
8.98
8.97
8.97
8.96
8.83
8.15
8.11
8.09
8.09
8.09
8.08
8.08
8.08
7.96
7.96
7.95
7.94
7.93
7.92
7.91
7.91
7.91
7.51
7.44
5.35
5.31
5.23
5.19
3.84
3.84
3.83
3.83
3.82
3.66
3.64
3.63
3.49
3.48
3.46
3.22
3.20
3.18
1.65
1.63
1.61
1.59
1.57
1.55
1.53
1.41
1.41
1.40
1.39
1.38
1.37
0.94
0.87
0.77
1.92
3.81
0.88
0.62
1.95
1.86
2.18
3.56
1.68
1.93
4.00
2.14
δ (ppm)

3 Protein production and purification

E. coli SE1 strains (Eurogentec) were transformed with pSCherry plasmids (Eurogentec) encoding the fusion Cherry-HD or Cherry-NLS-HD constructs (generous gift of Dr Alain Joliot). A $(His)_6$ tag was inserted at the N-terminus of the constructs and a PreScission protease site between the Cherry and Engrailed parts. Cells were grown in LB medium containing ampicillin (100 µg mL^{-1}) at 37 °C until reaching an absorbance at 600 nm of 0.8. IPTG was then added to the medium (final concentration 1 mM) and the bacteria were incubated at 37 °C for 3 h. The culture was centrifuged for 25 minutes at 6000 g at 4 °C. The pellet was taken up in lysis buffer (Table S1, 20–40 mL buffer per liter of culture), frozen in liquid nitrogen and kept at −20°C overnight. The pellet was unfrozen at 42 °C. Lysozyme (final concentration: 1 mg mL^{-1}) and protease inhibitor cocktail (PMSF, leupeptin, pepstatin) were added, and the mixture was incubated for 20 min at 4 °C. Sonication cycles were then performed (10 minutes; 1 cycle = 6 s at 70% maximum intensity and 6 s off). The mixture was then centrifuged at 30,000 g for 40 min at 4°C. The supernatant was filtered over 0.45 µm and 0.22 µm filters, and loaded on a HisTrap column (GE Healthcare). The protein was eluted with eluting buffer (Table S1). The collected fractions were pooled, and the fusion protein was cleaved overnight by Prescission protease at 4 °C in cleavage buffer. The mixture was then loaded on a His-Trap column. The non-retained fraction was collected and corresponded to the pure POI.

4 Protein labeling

4.1 *In vitro* labeling of Homeodomains

General procedure. A solution of protein in reaction buffer (100–200 µM) was degased and put under argon. 1 equivalent of TCEP was added, and the solution wad incubated for 30 min-1h at room temperature. 5 equivalents of [Re(CO)$_3$(Cl)(Pyta−maleimide)] were then added (stock solution in DMSO, final concentration of DMSO < 1% v:v), and the solution was incubated overnight at room temperature under inert atmosphere. Excess of reagent and TCEP were removed by repeated ultrafiltration (cut off 3kDa) with EDTA-free phosphate buffer. The resulting solution was analyzed by MALDI-TOF-MS, displaying one main peak corresponding to the desired product.

Reaction buffer: 50 mM phosphate buffer pH 6.5, 150 mM NaCl, 10 mM EDTA.

EDTA-free phosphate buffer: 50 mM phosphate buffer pH 7.0, 150 mM NaCl.

	MALDI-TOF-MS (SA matrix)	
Cl-HD	8117.71	[CysHD + Pyta]
Cl-NLS-HD	10062.16	[CysNLS−HD + Re(CO)$_3$Pyta]

4.2 Labeling of Carbonic Anhydrases with LDAI-SCoMPI

4.2.1 Stock solutions

Stock solutions of LDAI-SCoMPI reagents were done in DMSO and stored at −20°C. Concentrations were checked by measuring UV absorption at 280 nm or 335 nm.

4.2.2 *In vitro* labeling of human Carbonic Anhydrase 1 (hCA1)

A solution of hCA1 (10 µM in 50 mM HEPES pH 7.2 was incubated at 37°C for 6 h with 2 or 20 equivalents of LDAI-SCoMPI reagent. Every two hours, aliquots of the incubation solution were taken, treated and analysed either by MALDI-TOF MS or fluorescence gel imaging. A control experiment consisting in incubation solution + 10 equivalents EZA was performed for each experiment, and analysed at 6h.

Treatment for MALDI-TOF MS analysis: 10 µL of sample solution were quenched and purified using a ZipTip® (C18) and 0.1% aqueous TFA, then spotted on a MALDI plate. Peak areas of labeled and unlabeled protein were determined and percentage of labeling calculated.

Treatment for gel imaging analysis: 30 µL of sample solution were diluted with 30 µL 2X denaturing Laemmli buffer and stored at −30°C prior to gel analysis. Sample were heated at 70 °C for 20 min and loaded on a 12.5% SDS polyacrylamide gel. Fluorescence gel imaging was performed prior to CBB staining.

5 Imaging of SCoMPI-labeled proteins in cells

5.1 Stock solutions

Stock solutions of labeled homeodomains in EDTA-free phosphate buffer were stored at −20 °C. Their concentration was checked by measuring UV absorbance at 280 nm.

Stock solution of LDAI-SCoMPI: cf. Section 4.2.1.

5.2 Incubation conditions

5.2.1 SCoMPI-labeled homeodomains

Chinese Hamster Ovarian cells were seeded in 24-well plates containing 1.5mm×1.5mm×500nm Si_3N_4 membranes (purchased from Silson Ltd.), and grown for 24 h. Cells were washed once with HBSS buffer and once with fresh DMEM. Cells were then incubated with 10 µM solutions of protein in DMEM for one hour at 37 °C. Cells were washed twice with PBS, and incubated for 8 min with a 4% PFA solution in PBS at room temperature. Finally, cells were washed once with PBS and twice with milliQ water. Si_3N_4 slides were left to dry at air prior to examination by microspectroscopy.

5.2.2 SCoMPI-LDAI for the labeling of CA-IX and CA-XII

A549 cells were deposed in 24-wells plates in presence of Si_3N_4 slides (10^4 cells/well). After 24h, cells were put under hypoxic conditions for 24 h using Oxoid Anaerogen kit. Cells were then incubated with LDAI-SCoMPI reagents (10 µM) under normoxic conditions for 3 h at 37°C, washed 3 times with PBS and fixed with PFA (4 %)

5.3 Synchrotron X-ray Fluorescence microspectroscopy

Samples were examined on the new Nanoscopium beamline at SOLEIL synchrotron. Spectra and maps were recorded using a 13.5 keV incident beam energy. The X-ray beam was focused by a Kirckpatrick-Baez nano-focusing mirror to 0.3×0.3 μm^2 size at the sample position. The intensity of the focused X-ray beam was measured by a Si diode and was 6 e^{+09} photons/s. For the maps the FLYSCAN continuous scanning mode was used with 0.3 µm image pixel-size. The full XRF spectra were collected in each pixel by two Si-drift detectors (Ketek) in order to increase the solid angle of detection. Due to the varying Re concentrations the accumulation time/pixel depended on the proteins (Re-NLS-HD: 1.2 s/pixel, Re-HD: 4 s/pixel, CA: 1.8 s/pixel, EZA: 4.8 s/pixel) to decompose the overlapping Re and Zn peaks. The XRF spectra of the two detectors were added and the sum was used for calculating the elemental maps. A background masque was determined from the Ca and Zn intensity distributions for each scan and the corresponding average background spectrum per pixel has been calculated. This average background was then subtracted from the spectrum of each pixel of the scan. The obtained background corrected XRF spectra were processed in two different ways. On one hand, the sum-intensities were calculated within spectral regions (spectrum-ROI’s) of the identified elements by a home-made Matlab code and also by PyMCA. In Figure S8 the spectral region used for the Re-Lβ line-intensity is shown, as an example. Then the PyMCA software[6] was used for deconvoluting the overlapping Cu, Zn and Re peaks by batch fitting.

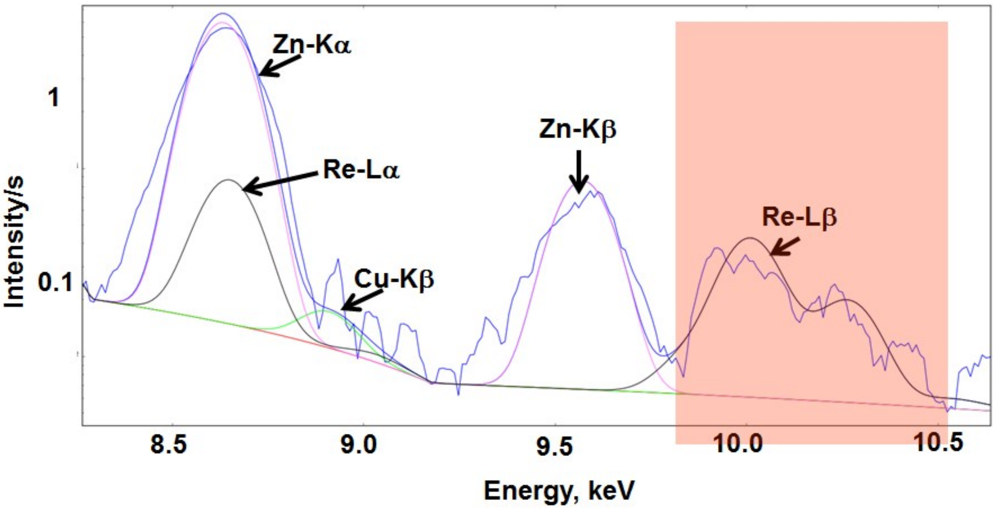

Figure S8. Cu, Zn and Re XRF lines in the mean spectrum of ROI5 of a Re-HD cell (shown in Fig S5). The figure shows the result of the PyMCA fit (green Cu, violet Zn and black Re) together with the measured data (blue). Only the spectral region in the vicinity of the Re L-lines is shown for clarity.

As can be seen in Figure S8, the Cu-Kβ, Zn-Kα and Re-Lα lines at ~8.6 keV are 100 % overlapping. However, the good energy resolution of the XRF detectors of the Nanoscopium beamline ensures the unambiguous deconvolution of the Zn-Kβ (9.6 keV) and the Re-Lβ lines (~10.15 and 10.28 keV). As such, in order to avoid the eventual Zn and Re signal overlapping in the Re images, we used only the Re-Lβ lines for creating the X-ray intensity maps. The Re maps obtained from the spectrum-ROI of the Re-Lβ line and from PyMCA spectrum fitting were carefully compared and in case of >3-sigma difference between the two values the XRF pixel spectra were investigated individually. In the Re images we show the values significantly exceeding the blank value (limit of blank or critical value[7], which is the Re intensity above the 1.65 x sigma value of the background intensity below the Re-Lβ line calculated from the background spectra. As an example, the XRF spectra in three different pixels of the Re-HD cells are shown in Figure S9. The red and blue spectra are two pixel-spectra within the cells and the green spectrum corresponds to a background spectrum.

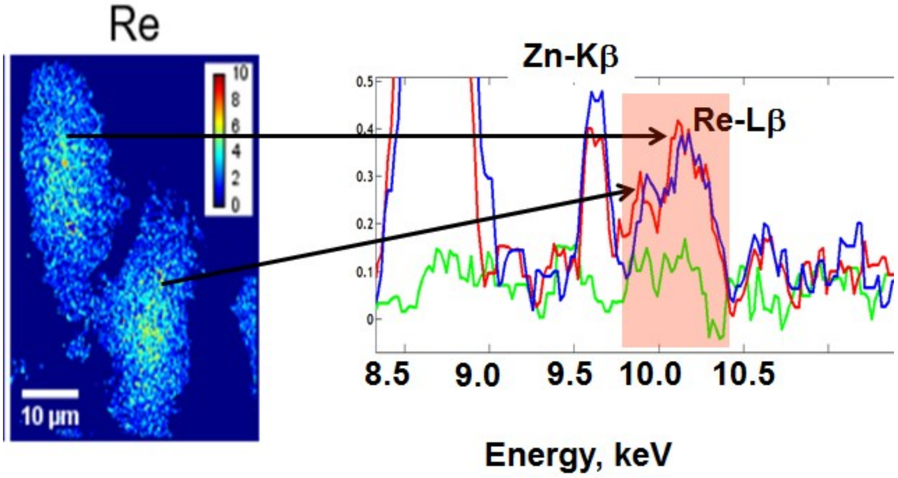

Figure S9. Background corrected XRF pixel spectra from the Re-HD cell map (shown in Fig S5). Only the spectral region in the vicinity of the Re L-lines is shown for clarity. The green spectrum is a background spectrum in a single pixel. The red and green lines show the XRF pixel spectra at the marked points of the map.

The calculated 3-sigma confidence level detection limit/pixel based on the Re-Lβ line is ~2.8 cps in the images. This means that in pixels having <2.8 cps intensity, Re is below the detection limit but it

exceeds the limit of blank. On the other hand the statistics of the mean spectra of chosen image areas, and as such the Re detection limit, is improving proportionally with the inverse of the square root of the number of summed individual pixels. As such, we checked and compared systematically the presence and statistical significance of Re by calculating the mean spectra of different ROI areas (e.g. in. Figure S5 and S6). These mean-spectra of the different cell ROI's were used for semi-quantification. The Re hot spots were removed from the Re map if necessary (with intensity > 50 counts/4 s for Re-HD and intensity > 29 counts/1.2 s for Re-NLS-HD). The RF7-200-S2372 thin film XRF Reference Sample (AXO Dresden GmbH) with 1 s acquisition time was measured in order to calibrate the geometrical factor for XRF semi-quantification. Moreover, 0.3 μL of a 10 μM solution of $Re(CO)_5Cl$ in MeOH was deposited on the silicon nitride membrane. The membrane was air-dried and XRF spectra were acquired for 300 s. The Re spectrum was fitted by PyMCA and was normalized to 1 s acquisition time for direct comparison with the normalized Re intensities extracted from the cell XRF-spectra. The semi-quantification provided ~1 x 10^{-21} mol 3-sigma confidence level detection limit for Re. The limit of quantification (≥ 6-sigma confidence[7]) was not reached for Re in our measurements due to the low statistics of the measured Re-Lβ signal.

6 References

[1] H. E. Gottlieb, V. Kotlyar, A. Nudelman, *J. Org. Chem.* **1997**, *62*, 7512–7515.

[2] V. Bonnard, S. Azoulay, A. D. Giorgio, N. Patino, *Chem. Commun.* **2009**, 2302–2304.

[3] M. van Dijk, C. F. van Nostrum, W. E. Hennink, D. T. S. Rijkers, R. M. J. Liskamp, *Biomacromolecules* **2010**, *11*, 1608–1614.

[4] A. Boulay, A. Seridi, C. Zedde, S. Ladeira, C. Picard, L. Maron, E. Benoist, *Eur. J. Inorg. Chem.* **2010**, *2010*, 5058–5062.

[5] K. Asano, S. Matsubara, *Org. Lett.* **2010**, *12*, 4988–4991.

[6] V. A. Solé, E. Papillon, M. Cotte, P. Walter, J. Susini, *Spectrochim. Acta Part B At. Spectrosc.* **2007**, *62*, 63–68.

[7] D. A. Armbruster T. Pry, *Clin Biochem Rev*. **2008**. Suppl 1, S49–S52.
